# Scalable One-Pot Production of Geranylgeranylated Proteins in Engineered Prokaryotes

**DOI:** 10.1021/acs.bioconjchem.4c00493

**Published:** 2025-03-03

**Authors:** Md Shahadat Hossain, Md Mahbubul Alam, Zhiwei Huang, Faeze Mousazadeh, Ronit Sarangi, Ebbing de Jong, Kavindu C. Kolamunna, Albert L. Adhya, James L. Hougland, Atanu Acharya, Davoud Mozhdehi

**Affiliations:** † Department of Chemistry, Syracuse University, 111 College Place, Syracuse, New York 13244, United States; ‡ Upstate Medical University, Proteomics and Mass Spectrometry, Weiskotten Hall 4307 WHA, 766 Irving Avenue, Syracuse, New York 13210, United States; § BioInspired Syracuse: Institute for Material and Living Systems, Syracuse University, Syracuse, New York 13244, United States

## Abstract

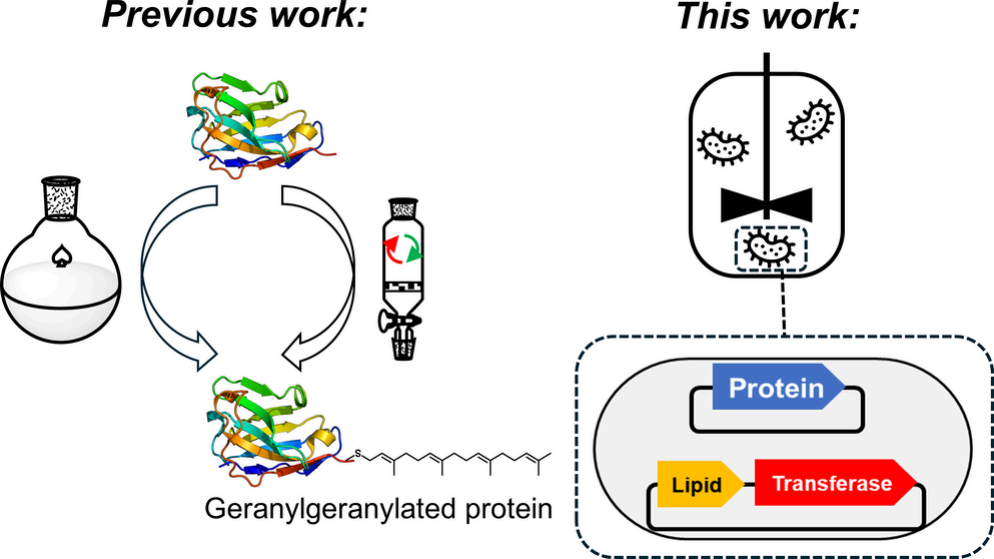

Geranylgeranylation is a critical post-translational modification essential for various cellular functions. However, current methods for synthesizing geranylgeranylated proteins are complex and costly, which hinders access to these proteins for both biophysical and biomaterials applications. Here, we present a method for the one-pot production of geranylgeranylated proteins in *Escherichia coli*. We engineered *E. coli* to express geranylgeranyl pyrophosphate synthase (GGS), an enzyme that catalyzes the production of geranylgeranyl pyrophosphate. By coexpressing GGS with a geranylgeranyltransferase, we achieved efficient geranylgeranylation of model protein substrates, including intrinsically disordered elastin-like polypeptides (ELPs) and globular proteins such as mCherry and the small GTPases RhoA and Rap1B. We examined the biophysical behavior of the resulting geranylgeranylated proteins and observed that this modification affects the phase-separation and nanoassembly of ELPs and lipid bilayer engagement of mCherry. Taken together, our method offers a scalable, versatile, and cost-effective strategy for producing geranylgeranylated proteins, paving the way for advances in biochemical research, therapeutic development, and biomaterial engineering.

## Introduction

Geranylgeranylation is a post-translational modification (PTM) involving the addition of a geranylgeranyl (20-carbon) isoprenoid group to proteins via a thioether linkage to a cysteine residue within the CaaX box motif near the C-terminus of protein substrates.^1^ The substrates for this PTM include many small GTPases, including members of the Rho, Rab, and Rap families, and γ subunits of heterotrimeric G proteins.^2^ Given its nonpolar nature, the geranylgeranylation is critical for membrane localization and function of the substrate proteins, especially their interactions with effector proteins and downstream cell signaling transduction.^3^ Therefore, dysregulation of geranylgeranylation and its biosynthesis has been linked to a wide range of human diseases, including cancer, type II diabetes, liver disorders, neurodegeneration, and others.^4,5^ Despite its significance, studying the role and mechanisms of this PTM and elucidating structure–function relationships in geranylgeranylated proteins (GG-proteins) has been hindered by the technical challenges of producing these lipid-modified proteins.^6,7^

Like other lipidated proteins, current methods for generating GG-proteins are labor-intensive, costly, and technically challenging.^8,9^ The primary challenge is that *Escherichia coli*, the workhorse organism for protein expression, lacks the enzymatic machinery required for GG-protein production.^10,11^ While eukaryotic or cell-free expression systems can overcome this limitation,^12−15^ they are expensive, difficult to scale, and often produce heterogeneously modified proteins in low yields.

To address these limitations, two primary strategies have been developed for geranylgeranyl modification of proteins. The first strategy involves semisynthetic approaches, in which recombinantly expressed proteins lacking the lipidated domain are conjugated to synthetic geranylgeranylated peptides using expressed protein ligation (EPL) or chemoenzymatic coupling.^16−19^ However, due to the acid lability of the geranylgeranyl group, solid-phase synthesis of geranylgeranylated peptides remains challenging, prompting the development of newer strategies that focus on late-stage chemoselective modifications of cysteine residues.^20−26^ Despite the complementary strengths of these approaches, both require significant reaction optimization and the use of organic solvents, detergents, and denaturants and an oxygen-free environment to balance the solubility and activity of proteins, reagents, and catalysts on a case-by-case basis. The high cost^27,28^ and limited stability of geranylgeranyl-based lipids further complicate efforts to efficiently produce GG-proteins at scale.^29,30^ Addressing these obstacles facilitates a deeper exploration of the biophysical implications of geranylgeranylation and enables the development of GG-modified proteins for advanced biomedical technologies and engineered materials.^31−33^

Here, we developed a one-pot method for producing GG-proteins in *E. coli*. To achieve this, we leveraged the endogenously produced isopentenyl diphosphate (IPP) and farnesyl pyrophosphate (FPP)—key intermediates in the biosynthesis of geranylgeranyl pyrophosphate (GGPP). We engineered *E. coli* to express geranylgeranyl pyrophosphate synthase (GGS), the enzyme that catalyzes GGPP formation, and geranylgeranyltransferase (GGT), which transfers GGPP to the CaaX motif of substrate proteins. For proof-of-concept, we tested our system using model proteins fused to a CaaX motif: intrinsically disordered elastin-like polypeptides (ELPs); globular proteins such as mCherry; and small GTPases including RhoA and Rap1B, which are native substrates of GGT. Our one-pot method demonstrated robust performance, achieving an isolated yield of 5 mg/L of culture, and was successfully scaled from 4 mL to 12 L in conventional lab settings, thus highlighting its potential for larger-scale applications.

## Results and Discussion

### Biochemical Characterization of a Bacterial GGS Enzyme

**Figure 1:**
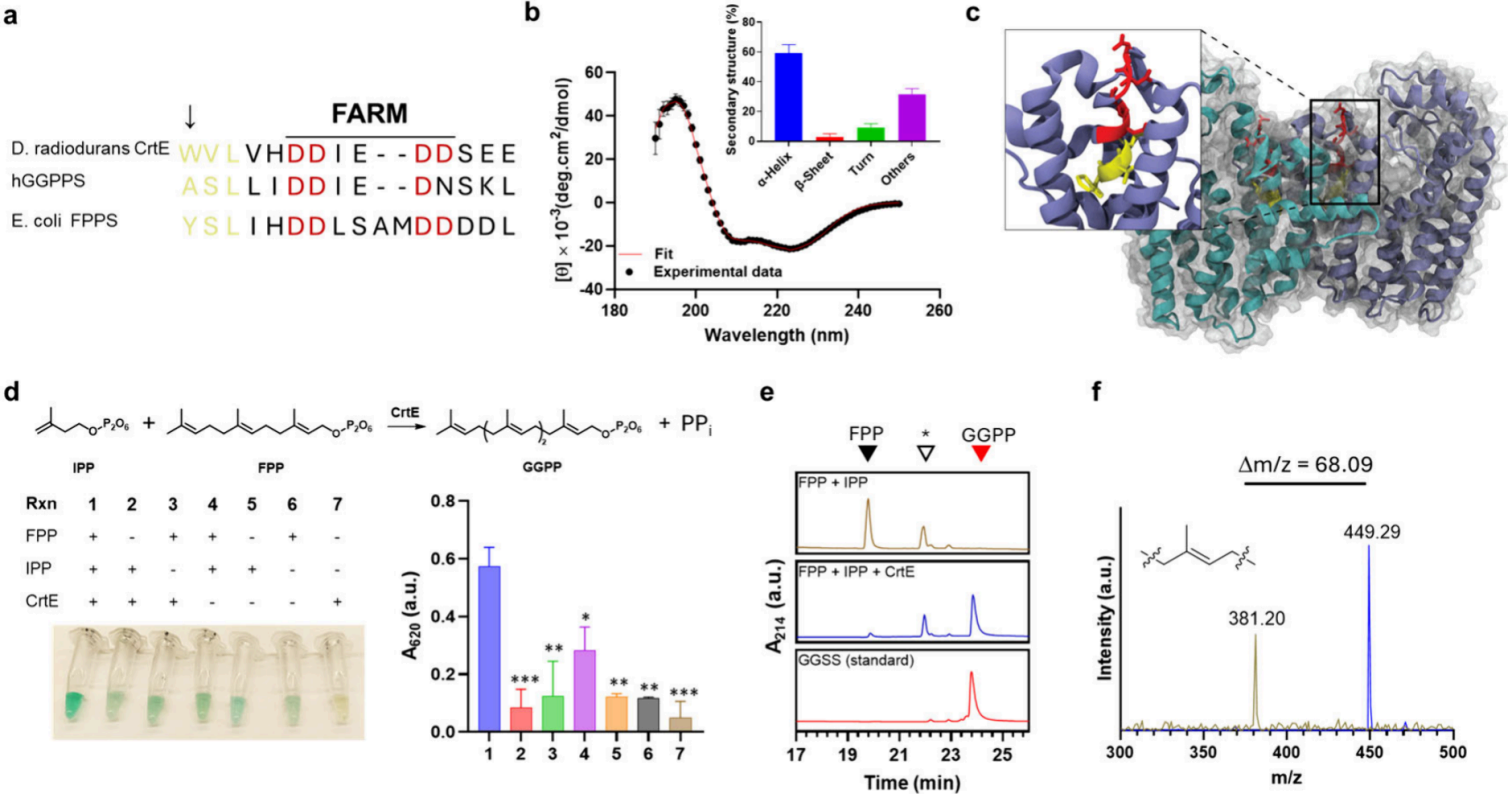
**Characterization of*D. radiodurans*geranylgeranyl pyrophosphate synthase (GGS).** (a) Sequence alignment of *D. radiodurans* CrtE with human and *E. coli* prenyl synthases, highlighting similarities in the first aspartic-rich motif (FARM, red) and the chain-length-determining region (CLD, yellow). (b) CD spectra of CrtE, revealing prominent α-helical content after deconvolution. (c) Representative structure of CrtE from MD simulations, highlighting its α-helical rich structure, with the FARM and CLD regions highlighted. (d) Schematic of the reaction between IPP and FPP to form GGPP and pyrophosphate, with quantification of the released phosphate using Biomol Green reagent. Combining FPP and IPP in the presence of CrtE results in a significant increase in absorbance at 620 nm, indicating the release of phosphate. (e) RP-HPLC chromatogram of reactions between FPP and IPP in the absence and presence of CrtE, showing a decrease in FPP peak intensity (black arrow) and the appearance of a new peak (red arrow) corresponding to GGPP. The asterisk denotes an impurity in FPP. (f) ESI-MS spectra of peaks labeled with black and red arrows, consistent with the molecular weight of FPP and GGPP, with mass shift indicating the addition of a C5 isoprenoid group. Error bars in (b, d) are std. dev. of *n* = 3. Statistical significance in (d) was determined using one-way ANOVA with Dunnett’s post hoc test. *p* < 0.05 (*), *p* < 0.01 (**), *p* < 0.001 (***).

To facilitate heterologous expression in *E. coli*, we aimed to identify a prokaryotic GGS enzyme, anticipating better compatibility with bacterial expression systems. We focused on a potential enzyme from *Deinococcus radiodurans*, an extremophilic bacterium known for producing a diverse array of carotenoids.^34^ The sequence of this protein (CrtE) showed a high degree of homology with the prenyl synthase family (Table S1), including a known FPP synthase (IspA in *E. coli*) and a human GGS. Interestingly, the *D. radiodurans* CrtE shares features with both eukaryotic GGPPS (the DDXXDD “first aspartic-rich motif (FARM)”) and prokaryotic enzymes (hydrophobic aromatic residues upstream of the FARM), which typically favor synthesis of shorter prenyl chains like FPP due to steric hindrance (Figure 1a, Figure S1). Given these mixed features and the lack of direct characterization, we prioritized in vitro studies to confirm the enzyme’s capacity to synthesize GGPP from *E. coli*’s endogenous FPP and IPP.

*First*, we synthesized a codon-optimized gene for the *D. radiodurans* GGS, incorporating an N-terminal his-tag for ease of purification (Tables S2 and S3). The protein was expressed and purified as detailed in the Materials and Methods (Figure S2). Circular Dichroism (CD) spectroscopy was used to assess the secondary structure of the purified enzyme, revealing a predominantly α-helical content (60 ± 6, Figure 1b). This is in line with the structure of other members of the prenyl synthase family and with predictions of AlphaFold3 and subsequent MD simulations (75 ± 1, Figure 1c, Figures S3–S6).^35^*Next*, we validated that the purified protein catalyzes formation of GGPP, following the reaction scheme depicted in Figure 1d. Reaction progress was monitored using two methods: a colorimetric end-point assay to detect the release of pyrophosphate (PPi),^36^Figure 1d; and reverse-phase high-performance liquid chromatography (RP-HPLC) with mass spectrometry to directly detect GGPP formation (Figure 1e,f). We observed a significant increase in absorbance at 620 nm, indicating the release of phosphate, only when all three components of the reaction (FPP, IPP, and CrtE) were present. RP-HPLC further confirmed the formation of GGPP, as indicated by the appearance of a new peak corresponding to GGPP, benchmarked against chemically synthesized GGPP (bottom trace; Figure 1e). *Finally*, electrospray ionization mass spectrometry (ESI-MS) further verified the reaction product, revealing a mass increase of 68.09 Da, consistent with the addition of a prenyl group. The observed mass of 449.1844 Da aligns with the theoretical mass of [GGPP-H]^−1^ (449.1858 Da). Together, these experiments validate that *D. radiodurans* CrtE is capable of synthesizing GGPP from FPP and IPP, providing a solid foundation for its use as the GGS enzyme in our one-pot method for producing geranylgeranylated proteins.

### Validating GG-Protein Production in *E. coli* Engineered to Express GGS and GGT

**Figure 2:**
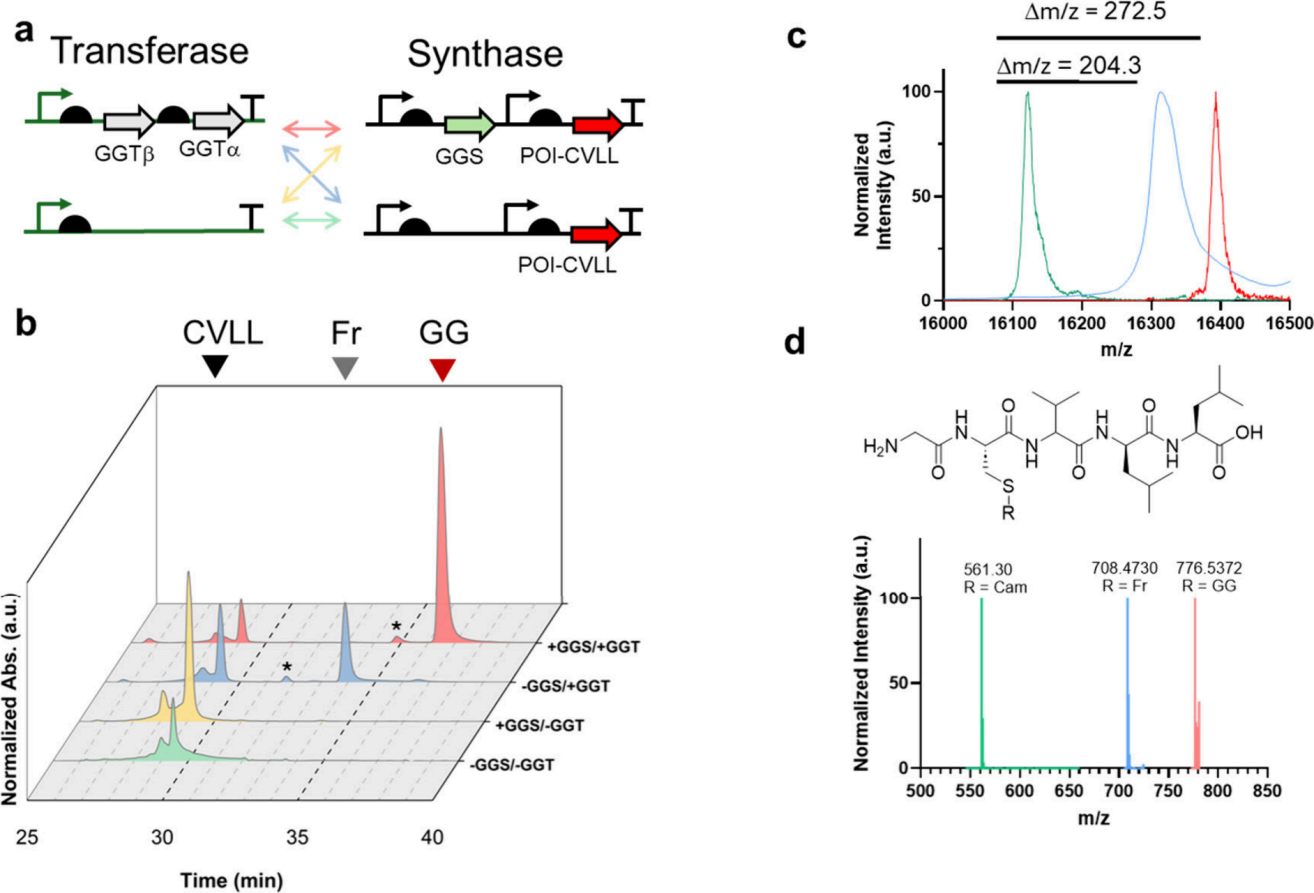
**Plasmid architecture and analysis of geranylgeranylated model proteins.** (a) Schematic of the plasmid architecture used in this study, featuring two orthogonal plasmids for coexpression of the protein of interest with GGS and the two subunits of GGT. Control plasmids are utilized to evaluate the effects of GGS and GGT in a 2 × 2 factorial experiment. (b) RP-HPLC chromatogram of a model protein coexpressed in four strains (±GGS/ ±GGT). In the absence of GGT, only the unmodified protein and its disulfide-bonded dimer are detected. Expression of GGT results in the modification of the protein with a hydrophobic motif, depending on whether GGS is coexpressed. Peaks marked with an asterisk (*) are assigned to the oxidized (sulfoxide) form of the lipidated protein. (c) MALDI-TOF spectra of unmodified and hydrophobically modified protein isoforms, showing differences in molecular weight that suggest modification with one farnesyl group in the (−GGS/+GGT) strain and one geranylgeranyl group in the (+GGS/+GGT) strain. (d) Mass spectra of trypsin-digested proteins confirm the proposed modifications and establish the site of lipidation to the CaaX box sequence. Carboxymethylacetamide (Cam) is used for alkylating free thiols of the unmodified constructs to prevent dimerization.

Building on the validation of GGS, we next established a minimalistic yet efficient system for geranylgeranylation in *E. coli*. This system necessitated the coexpression of two critical components, GGS and GGT. We opted to use a type-I GGT enzyme from *Rattus norvegicus* due to its broad substrate scope and ability to modify proteins with a CaaX box motif at their C terminus.^37^ Since GGT is a heterodimer, we used two orthogonal plasmids to coexpress four polypeptide chains: the α and β subunits of GGT, GGS, and the protein substrate fused to the model CaaX box motif, CVLL (Figure 2a). To address the aggregation tendency of the α subunit, we employed a translationally coupled expression system, while an orthogonal bicistronic plasmid was used to express GGS and the substrate in commonly used *E. coli* BL21(DE3) strains. Control vectors lacking either GGT or GGS were also designed to systematically evaluate the efficiency of GG-protein production (Table S3). Pairwise combinations of these vectors enabled a systematic assessment of our one-pot GG-protein production system (Table S4).

As our first model protein, we selected an elastin-like polypeptide (ELP), an artificial intrinsically disordered protein derived from the consensus sequence of tropoelastin.^38^ ELP’s disordered and uniquely hydrophobic nature allows facile isolation by treating the cells with isopropanol,^39^ which results in selective partitioning of the ELP from the rest of the proteome. To evaluate the efficacy of our engineered *E. coli* strains in facilitating protein geranylgeranylation, we isolated the model protein from each strain and analyzed its isoforms using RP-HPLC (Figure 2b) and matrix-assisted laser desorption ionization time-of-flight mass spectrometry (MALDI-TOF-MS, Figure 2c). ELP expressed in the absence of GGT, i.e., in -GGS/-GGT and +GGS/-GGT strains, had similar retention times on the HPLC traces, showing two prominent peaks corresponding to the unmodified protein (t_R_ = 29.0 ± 0.1 min) and the disulfide-bonded dimer (t_R_ = 29.4 ± 0.1 min), Figure S7. MALDI-TOF-MS analysis confirmed these findings, as observed *m*/*z* for these constructs closely matched the molecular weight of the unmodified ELP. Introduction of GGT alone (-GGS/+GGT strain) led to the appearance of a new species with an elution time of 33.9 ± 0.1 min (blue trace, Figure 2b), which was confirmed to be a farnesylated (Fr) ELP product based on the results of MALDI-TOF-MS (blue line, Figure 2c). Given that GGT can accept FPP in the absence of GGPP, this reaction condition leverages *E. coli*’s endogenous FPP supply, albeit with low yield as only 50% of the expressed ELPs were farnesylated (Figure 2b).^40^ Importantly, the strain coexpressing both GGS and GGT (+GGS/+GGT) produced a distinct peak at 36.5 min on the RP-HPLC chromatogram (red trace, Figure 2b), which was confirmed to be GG-protein by MALDI-TOF (+272.5 peak, Figure 2c). Under these conditions, 90% of the expressed ELPs were modified with GG, and no sign of a farnesyl-modified ELP was observed on the HPLC trace (Figure 2b). Finally, trypsin digest and subsequent LC-MS/MS analyses provided robust evidence that the modifications—whether farnesyl or geranylgeranyl—were specifically localized to the cysteine residue of the CaaX box (Figure 2d, Figure S8, and Table S5). Additionally, LC-MS analysis detected a small fraction of oxidized (sulfoxide) peptide, consistent with aerobic oxidation of the thioether moiety (Figures S9 and S10). Collectively, these results indicate that our engineered *E. coli* system produces GG-protein with a high efficiency.

### Biophysical Characterization of ELPs with Geranylgeranyl Modification

To showcase the versatility of our one-pot method for GG-protein production, we leveraged it to rapidly generate a diverse library of prenylated ELPs (Figures S11, S12, Tables S6–S8). In contrast to traditional chemoenzymatic approaches, which require laborious reaction optimization for each protein, our genetically encoded process simplifies the production by merely controlling the producer strain, making it highly accessible to standard cultivation technologies. This streamlined process accelerates the production of modified proteins at scale. Using our engineered strains, we efficiently produced nine proteins, encompassing three ELP variants with different lengths and hydrophobicities, each in unmodified, farnesyl-modified (-Fr), and geranylgeranyl-modified (-GG) isoforms. This capability allowed us to systematically examine how lipid modifications influence the phase behavior of intrinsically disordered ELPs, revealing significant effects of prenylation on their biophysical properties.

**Figure 3:**
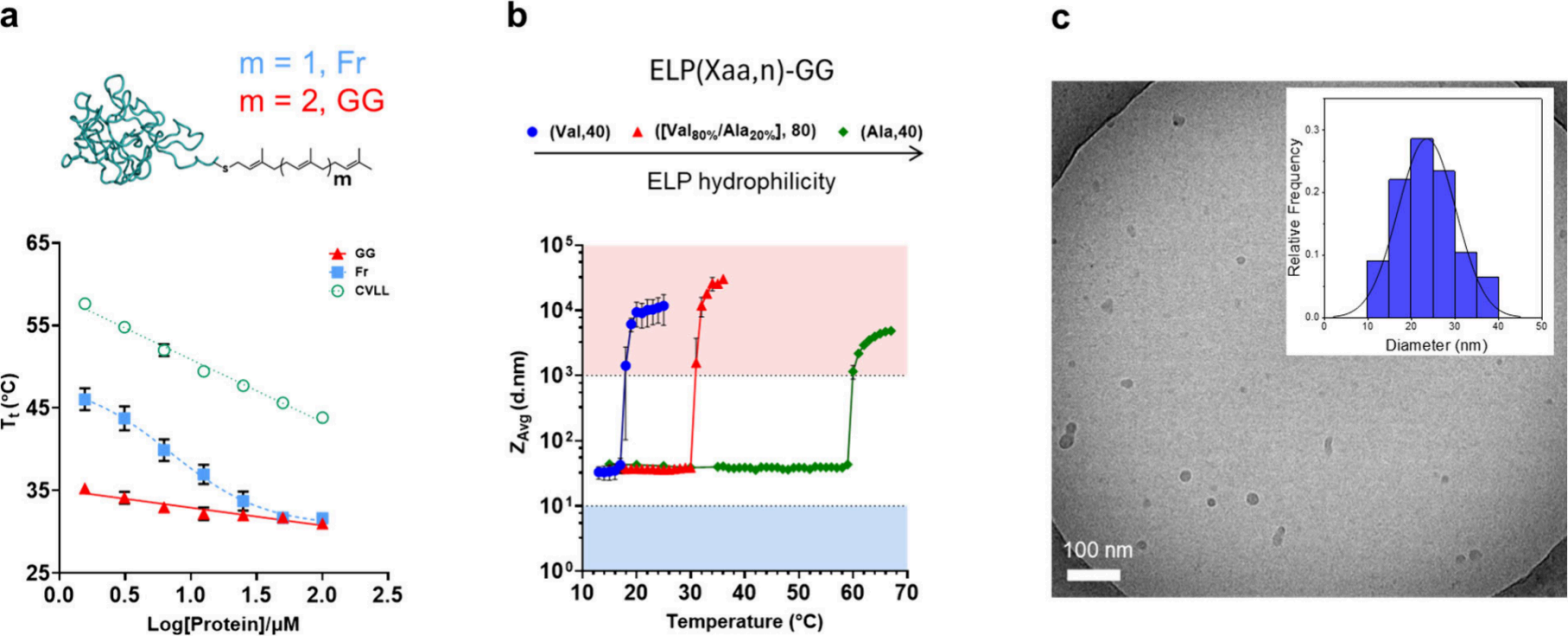
**Biophysical impact of GG-modification on model disordered proteins.** (a) Representative temperature–composition phase diagrams for unmodified, Fr-, and GG-modified ELP (V8/A2)_80_. Prenylation alters the phase boundaries, with the extent of this alteration being dependent on the nature of the attached lipid. (b) Variable-temperature DLS confirms that GG-modified ELPs form stable nanoparticles across all tested variants, with a sharp micelle-to-coacervate transition at higher temperatures. Blue, white, and red regions represent unimers, micelles, and coacervates, respectively. (c) Representative cryo-TEM visualization of GG-modified ELPV_40_ nanoparticles, with the inset displaying the nanoparticle diameter histogram. Error bars in (a, b) are standard deviations of three measurements. See Figures S14–S16 for data for other ELP variants.

Both Fr- and GG-modification increased the propensity of ELPs to phase-separate by lowering their phase boundaries compared to unmodified isoforms, with the degree of reduction dependent on the physicochemistry of the lipid (i.e., its length) (Figure 3a, Figure S13, Figure S14). The transition temperature versus the natural log of concentration for both unmodified and GG-modified ELPs followed a linear model, with GG-modified constructs exhibiting a significantly lower slope and intercept compared to unmodified proteins (Tables S9, S10). In contrast, Fr-modified proteins followed a sigmoidal model, where their behavior at high concentrations was similar to GG-modified proteins, but at lower concentrations, they displayed intermediate behavior between GG-modified and unmodified isoforms. These findings are important for two reasons: First, they demonstrate that prenylation can modulate the phase behavior of proteins, adding to an emerging body of literature showing that lipidation can regulate the phase separation and material properties of protein condensates.^41−44^ Given the prevalence of prenylation, this offers new insights into how such modifications affect protein behavior in cellular environments. Second, the distinct behavior of GG-modified ELPs—with reduced variations in transition temperature across a broad concentration range—suggests that the increased hydrophobicity of the GG lipid leads to a more stable and pronounced alteration in the quaternary organization of the ELP chains.^45^

Consistent with the turbidimetry, dynamic light scattering (DLS) revealed that geranylgeranylation leads to the formation of stable, thermoresponsive nanoparticles across all tested ELP variants (Figure 3b, Figure S15). The size of these GG-modified nanoparticles remained consistent, with hydrodynamic radii of 40 ± 5 nm, regardless of the ELP composition or length—a 40-mer with Valine, a 40-mer with Alanine, or a mixed 80-mer with 80% Valine and 20% Alanine as guest residues, Table S11. This behavior was further supported by cryo-TEM (Figure 3c, Figure S16), which confirmed the formation of spherical nanoparticles of similar dimensions. Importantly, while the nanoparticle size remained independent of ELP composition, all GG-modified nanoparticles underwent a sharp increase in size at elevated temperatures, consistent with coacervation. This micelle-to-coacervate transition temperature was tunable by altering the composition of the ELPs. This decoupling of nanoparticle size from the transition temperature represents a significant advantage over systems modified with saturated fatty acids, where changes in ELP composition not only alter their LCST but also significantly impact the size and shape of the assemblies.^46−48^ The ability to maintain a stable nanoparticle size while fine-tuning the transition temperature improves the programmability of GG-modified constructs, enhancing their potential for drug delivery and tissue engineering applications.^49^

### Validating the One-Pot Method for Geranylgeranylation of Globular Protein

**Figure 4:**
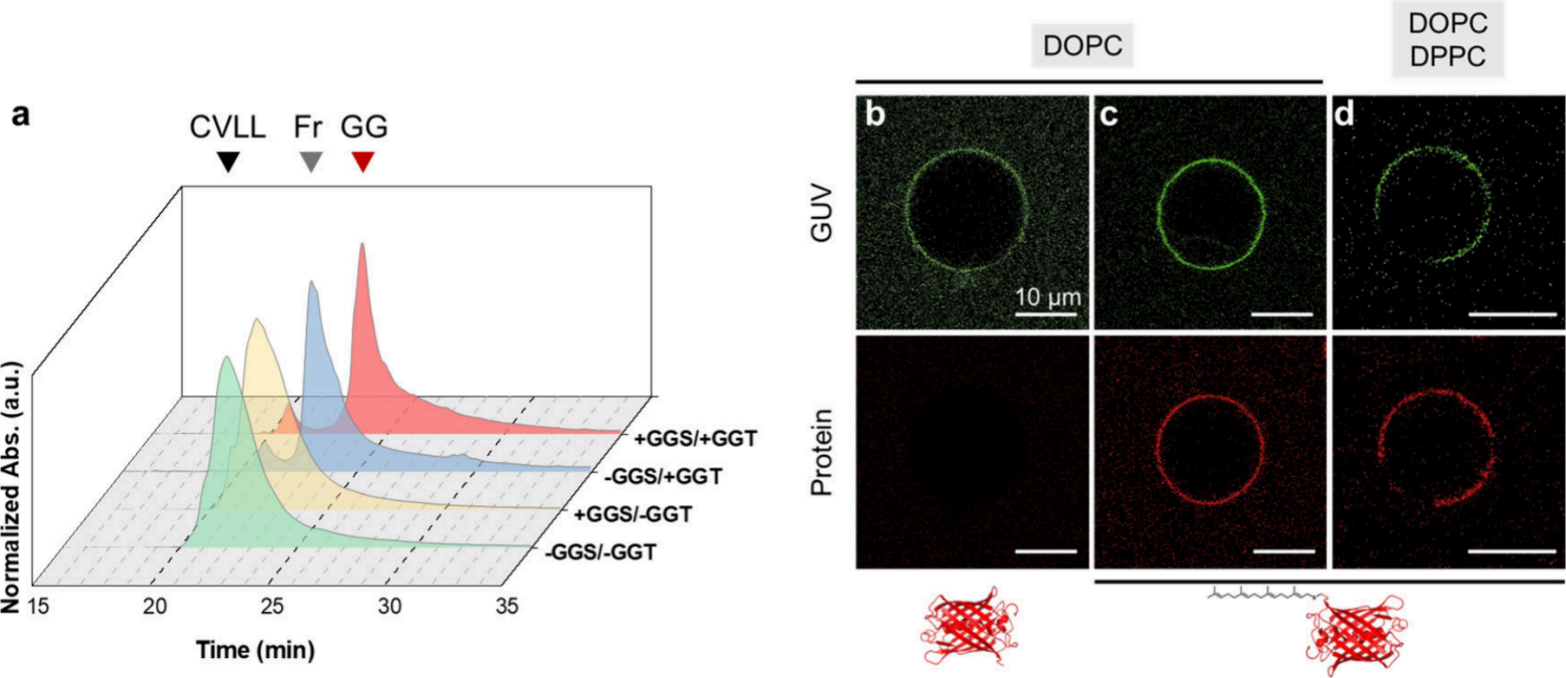
**One-pot GG-modification of mCherry and visualization of its interaction with model membranes.** (a) RP-HPLC traces of mCherry expressed in ±GGS/ ±GGT strains, demonstrating the applicability of the method to globular proteins. (b, d) Confocal microscopy images showing interactions between unmodified and GG-modified mCherry with model GUVs. Geranylgeranylation enhances mCherry’s interaction with GUVs, promoting colocalization into lipid-disordered (l_d_) regions.

To demonstrate the generalizability of our recombinant method for protein lipidation, we applied it to a globular fluorescent protein, monomeric red fluorescent protein (mCherry). Our mCherry construct included an N-terminal His-tag for purification and a C-terminal CVLL peptide for lipidation. Consistent with results obtained with ELPs, no lipidation was observed in the absence of GGT. When GGT was present, the attached lipid was determined by the coexpression of GGS. Without GGS, the expressed protein was primarily farnesylated; with GGS, more than 95% was geranylgeranylated (Figure 4a, Figure S17,S18). Notably, mCherry-GG remained soluble and did not strongly associate with *E. coli* membrane fractions, and its fluorescence spectrum was indistinguishable from that of the unlipidated construct (Figure S19), which confirms that recombinant geranylgeranylation did not impair the protein’s folding or function. This result is significant because conventional methods for geranylgeranylation often require surfactants or denaturants,^24^ which necessitate extensive downstream processing to remove these agents and refold the proteins, often with uncertain recovery of function.

Further analysis revealed that geranylgeranylation enhanced mCherry’s ability to associate with lipid bilayers, with the anchored mCherry showing a preference for lipid-disordered domains. Incubation of unmodified mCherry with giant unilamellar vesicles (GUVs) prepared from 1,2-dioleoyl-*sn*-glycero-3-phosphocholine (18:1 (Δ9-Cis) PC, DOPC) did not result in substantial interaction between the protein and the GUVs (Figure 4b). In contrast, mCherry-GG readily associated with DOPC GUVs, exemplified by the colocalization between the fluorescence channel (red) and the GUVs (Figure 4c), with no detectable changes in the GUV morphology. When GUVs were prepared with a mixture of saturated and unsaturated phospholipids (e.g., DPPC and DOPC), the mCherry-GG interacted with GUVs but preferentially colocalized with the regions containing unsaturated lipids (DOPC) (Figure 4d). This behavior aligns with the physical properties of the geranylgeranyl lipid, which contains unsaturated bonds and favors interactions with lipid-disordered regions.^50^

Finally, to demonstrate the applicability of our approach for producing natively geranylgeranylated proteins, we cloned two small GTPases, RhoA (a prototypical member of the Rho family involved in cytoskeletal dynamics) and Rap1B (a Ras-related protein that counteracts oncogenic Ras mutants). As shown in Figures S20 and S21, our strains efficiently geranylgeranylated both proteins, achieving >95% modification for RhoA and 87% for Rap1B as determined by RP-HPLC. These findings are particularly noteworthy because, consistent with previous reports,^51^ even the nonlipidated recombinant Rap1B accumulated in inclusion bodies. Achieving a high modification efficiency under these suboptimal conditions highlights the robustness and broad applicability of our approach.

LC-MS analysis confirmed modification of the C-terminal peptide fragments (Figures S22 and S23) and identified oxidation at the thioether bond (Figure S24), affecting 22% of RhoA and 5% of Rap1B as determined by HPLC. Thioether oxidation, similar to methionine oxidation,^52^ is well-documented and has been observed in vitro for prenylated peptides.^53^ While the precise source of this oxidation remains unclear in our system, we attribute it primarily to air exposure during purification. To mitigate this, we propose optimizing the purification protocol, such as degassing buffers, to minimize oxidation. Additionally, to address the presence of residual unmodified protein, we propose using hydrophobic interaction chromatography^54^ or surfactant-based phase separation^55^ to selectively isolate lipidated proteins from their unmodified counterparts, providing scalable solutions for producing high-purity geranylgeranylated proteins suitable for biochemical and structural studies.

## Conclusions

The challenging synthesis of lipidated proteins remains a key barrier to understanding how lipidation affects protein structure and function at the molecular level, despite the well-established role of this PTM in regulating cellular processes. This challenge also limits lipidation’s potential as a tool for engineering protein behavior and designing functional materials. Here, we addressed these obstacles by developing a robust, user-friendly system for producing GG-modified proteins in *E. coli*. Our one-pot method enables efficient geranylgeranylation of proteins without compromising their structural integrity or function. The ability to geranylgeranylate a structurally demanding protein like mCherry, while preserving its function, highlights the robustness and versatility of our approach. This method offers significant advantages over conventional lipidation techniques, by eliminating the need for harsh chemicals and complex refolding steps. The isolated yields for geranylgeranylated proteins in this study was 5 mg/L of culture (except for ELPA_40_-GG, which yielded ∼1 mg/L), even without optimization of expression conditions, codon usage, or plasmid design. These results highlight the baseline efficiency of our system, and we anticipate that targeted refinements—such as tuning induction parameters or enhancing translational efficiency—could substantially improve the production yield of GG-proteins.

The complete genetic encoding of our platform unlocks exciting avenues for future research such as the evolution of *E. coli* strains capable of synthesizing and transferring non-natural isoprenoid analogues—lipid molecules not typically found in nature.^56,57^ The unique physicochemistry of these noncanonical lipids can then be exploited to precisely control lipidated protein behavior, such as membrane affinity, subcellular localization, and protein–protein interactions.^58−60^ Additionally, many prenylated proteins undergo further post-translational modifications after prenylation—such as endoproteolytic processing of the CaaX motif, followed by carboxyl methylation—before reaching their mature, functional forms.^61^ Recognizing the critical role of these modifications in proper protein function and localization, we are engineering strains to support these processing steps within a prokaryotic system, enabling the efficient production of fully mature, post-translationally modified proteins. Ongoing research explores how GG’s unique physicochemical properties can regulate the pharmacokinetics, internalization, and intracellular distribution of protein therapeutics. Overall, our recombinant platform provides a reliable and scalable approach to producing prenylated proteins, overcoming previous production challenges and paving the way for advancements in lipoengineering, synthetic biology, biomedical engineering, and materials science.

## Data Availability

The data supporting this article has been included as part of the Supporting Information.

## Supporting Information Available

Detailed Materials and Methods, Supplementary Tables, Supplementary Figures (PDF)
