# Supplementary material for: Scalable One-Pot Production of Geranylgeranylated Proteins in Engineered Prokaryotes
Source: Bioconjug Chem. 2025 Mar 3;36(3):415–23. doi: 10.1021/acs.bioconjchem.4c00493 (PMC11926785; doi:10.1021/acs.bioconjchem.4c00493)
Supplement: Supplementary file 1 — bc4c00493_si_001.pdf [file bc4c00493_si_001.pdf]

Supplementary Information

Scalable One-Pot Production of Geranylgeranylated Proteins in Engineered Prokaryotes

*Md Shahadat Hossain, Md Mahbubul Alam, Zhiwei Huang, Faeze Mousazadeh, Ronit Sarangi, Ebbing de Jong, Kavindu C. Kolamunna, Albert L. Adhya, James L. Hougland, Atanu Acharya, Davoud Mozhdehi**

dmozhdeh@syr.edu

Table of Contents

1. Materials S3
2. Cloning S3
   2.1 General procedures S3
   2.2 Cloning of codon-optimized GGS in pET24a S4
   2.3 Cloning of ELP-CVLL variants in pET24a S4
   2.4 Construction of pACYCDuet vectors S4
      2.4.1 Representative cloning method used for ELPs S5
      2.4.2 Modified procedure for mCherry S5
   2.5 Construction of pET23 vectors S6
      2.5.1 pET23_GGTβ_GGTα S6
      2.5.2 pET23_Control S6
3. SDS-PAGE and western blot analysis S7
4. RP-HPLC S7
5. MALDI-TOF S8
6. Trypsin digestion and LC-MS S8
7. Protein expression and purification S9
   7.1 Expression and purification of 6xHis-GGS S9
   7.2 Expression and purification of ELP Isoforms S10
   7.3 Expression and purification of mCherry Isoforms S11
   7.4 Expression and purification of RhoA Isoforms S12
   7.5 Expression and purification of Rap1B Isoforms S13
8. Circular dichroism S14
9. Computational modelling S14
   9.1 System building S14
   9.2 MD simulation S15
   9.3 GaMD simulation S15
10. GGS validation assay S15
11. VT-Turbidimetry S17
12. Dynamic light scattering S17
13. Cryo-TEM S18
14. Membrane interaction studies S18
15. Supplementary tables S20
16. Supplementary figures S27
17. References S51

1. Materials

The chemically competent NEb5alpha, BL21(DE3) and T7 Express cells, restriction enzymes, ligase, and corresponding buffers and DNA extraction kits were purchased from New England Biolabs (Ipswich, MA). Isopropyl β-D-1-thiogalactopyranoside (IPTG), Apomyoglobin, Cytochrome C, Aldolase, sinapinic acid, zinc sulfate, and Trifluoroacetic acid (TFA) were purchased from Sigma Aldrich (St. Louis, MO). Tryptone, yeast extract, sodium chloride, ampicillin, kanamycin, phosphate buffer saline (PBS), DMSO, isopropanol, acetonitrile, ethanol, 6x-His Tag Monoclonal Antibody (HIS.H8); Alexa Fluor™ 488, NBD Cholesterol [22-(N-(7-Nitrobenz-2-Oxa-1,3-Diazol-4-yl) Amino)-23,24-Bisnor-5-Cholen-3β-Ol] and bovine serum albumin (BSA) were obtained from ThermoFisher Scientific (Waltham, MA). High performance liquid chromatography (HPLC) grade acetonitrile was purchased from Fisher Scientific (Fair Lawn, NJ). Chloramphenicol was purchased from BIOBASIC (Markham, Canada). Mini-PROTEIN TGX stain free precast gels, precision plus protein unstained protein standards, 0.2 μm nitrocellulose membrane, Trans-Blot Turbo Transfer pack and EveryBlot Blocking Buffer were purchased from Bio-Rad Laboratories, Inc. (Hercules, CA). Deionized water was obtained from Milli-Q system (Millipore SAS, France). Simply Blue™ Safe stain was purchased from Novex (Van Allen Way Carlsbad, CA). 1-palmitoyl-2-oleoyl-sn-glycero-3-phosphocholine (POPC) and 1,2-dioleoyl-sn-glycero-3-phosphocholine (DOPC) were purchased from Avanti Polar Lipids (Alabaster, AL, USA). All chemicals were used as received without further purification. All oligonucleotides and gene fragments were purchased from Integrated DNA Technologies.

2. Cloning

2.1 General procedures

The genes and plasmids used in this study were constructed using standard molecular biology techniques, as detailed in the sections below. Non-repetitive genes (e.g., GGS, mCherry) were ordered as gene fragments from IDT DNA and assembled into vectors using NEBuilder® HiFi DNA Assembly according to the manufacturer's instructions. Gene amplification was performed using Q5 High-Fidelity Hot Start polymerase, with annealing temperatures optimized by gradient PCR (± 5°C of the predicted annealing temperature by NEB Tm Calculator). Site-directed mutagenesis was performed using the Q5 Site-Directed Mutagenesis Kit with primers designed via NEB BaseChanger, and gradient PCR was applied as needed to optimize reaction conditions. Repetitive sequences (e.g., ELP variants), which are difficult to amplify due to degenerate codons, were assembled using recursive directional ligation by plasmid reconstruction (PRe-RDL) and cloned into final vectors using restriction enzyme-based ligation. All constructs were verified by Sanger sequencing (Genewiz), and final plasmids were fully sequence-verified using nanopore sequencing (Plasmidsaurus).

2.2 Cloning of codon-optimized GGS in pET24a

For the in vitro validation of *Deinococcus radiodurans* crtE (i.e., GGS), the codon-optimized cDNA was ordered as gene fragments from IDT, in frame with an N-terminal 6xHis tag and flanked by ~20 bp homology regions matching the pET24a vector. The pET24a vector (1 µg) was linearized by double digestion with *NdeI* and *BamHI-HF*, followed by purification through 1% agarose gel electrophoresis (130 V, 40 min) and gel extraction using the NEB Monarch Kit.

The linearized vector (50 ng) and synthetic gblock insert were combined at a 1:2 vector-to-insert ratio and assembled using NEBuilder HiFi master mix (50°C, 1 hour). After assembly, 2 µL of the reaction mixture was transformed into chemically competent E. coli Eb5α cells using heat shock (42°C, 30 seconds) and plated on LB agar containing 45 µg/mL kanamycin. Colony PCR was performed on selected colonies using standard T7 promoter primers, and positive constructs were confirmed by DNA sequencing.

2.3 Cloning of ELP-CVLL variants in pET24a

Fusion of ELP variants to the CVLL peptide, a substrate for geranylgeranyl transferase (GGT), was accomplished using a previously established procedure.[1] Phosphorylated sense and antisense oligonucleotides encoding the CVLL sequence were annealed at 95°C for 5 minutes, followed by cooling to room temperature in annealing buffer (50 mM Tris-HCl, 10 mM $MgCl_2$, 1 mM ATP, 10 mM DTT, pH 7.5). The annealed product was ligated into a modified pET24a vector, which had been linearized by double digestion with *BseRI* and *BamHI* and purified by agarose gel extraction. The ligation reaction, carried out using a 1:3 vector-to-insert ratio with a quick ligation kit, was incubated at room temperature for 15 minutes. The ligation products were transformed into chemically competent E. coli Eb5a cells using the heat shock method (42°C, 30 seconds). Transformed cells were plated on LB agar containing 45 µg/mL kanamycin, and positive constructs were identified by sequencing with the T7-term primer.

Next, the recursive directional ligation by plasmid reconstruction (PRe-RDL) method was employed to fuse the CVLL peptide sequence to the C-termini of ELP variants.[2] The ELP plasmid (1 µg) was digested with *AcuI* and *BglI*, while the CVLL plasmid (1 µg) was digested with *BseRI* and *BglI* for 3 hours at 37°C. DNA fragments were separated on a 1% agarose gel (130 V, 40 minutes), and the desired ELP and CVLL fragments were excised based on their predicted sizes, followed by purification using the Monarch Gel Extraction Kit (NEB).

2.4 Construction of pACYCDuet vectors

For in vivo geranylgeranylation studies, the pACYCDuet vector was used to co-express GGS and model proteins fused to the CVLL sequence. The construction of these vectors involved two steps: (1) subcloning of the GGS gene into the first multiple cloning site of the pACYCDuet vector, and (2) subcloning of POI-CVLL fusion genes into the second multiple cloning site.

2.4.1 Representative cloning method used for ELPs

First, the GGS gene was amplified from the pET24a plasmid using GGS-F and GGS-R primers (**Table S2**) in a PCR reaction containing 3 ng of template DNA, 500 nM of each primer, and Q5 High-Fidelity Hot Start master mix (NEB). The PCR conditions were initial denaturation at 98°C for 30 s, followed by 30 cycles of 98°C for 10 s, annealing at 70 °C for 30 s, and extension at 72°C for 30 s, with a final extension at 72°C for 2 minutes. The PCR products were purified using the Monarch PCR & DNA Cleanup Kit (NEB). The pACYCDuet-1 recipient vector was linearized by double digestion with *AscI* and *NcoI-HF*, and the linearized vector was gel-purified. The purified GGS PCR product and linearized vector were combined at a 1:2 molar ratio and assembled using NEBuilder HiFi Master Mix (50°C, 30 minutes). The assembled plasmid (2 µL) was transformed into 25 µL of chemically competent NEB5α cells by heat shock and plated on LB agar containing 25 µg/mL chloramphenicol. Positive colonies were identified by colony PCR using ACYCDuetUP1 and DuetDown-1 primers and confirmed by sequencing.

Next, the ELP-CVLL gene was excised from the donor pET24a plasmid using *NdeI* and *XhoI* and gel-purified. The recipient pACYCDuet_GGS vector was linearized by digestion with *NdeI* and *XhoI*, and the linearized vector and ELP-CVLL insert were ligated in a 1:3 molar ratio using a quick ligation kit at room temperature for 10 minutes. The ligation mixture (3 µL) was transformed into 25 µL of NEB5α cells by heat shock and plated on LB agar containing 25 µg/mL chloramphenicol. Positive constructs (pACYDuet_GGS_POI-CVLL) were confirmed by sequencing.

For the construction of control pACYCDuet plasmids (lacking GGS), the first step of the procedure was skipped, and ELP-CVLL was directly cloned into an unmodified pACYCDuet vector (resulting in pACYDuet_()_ELP-CVLL plasmids).

2.4.2 Modified procedure for mCherry

To facilitate the purification of mCherry isoforms using immobilized metal affinity chromatography (IMAC), the following modifications to the cloning workflow were implemented: First, Q5 site-directed mutagenesis (Q5-SDM) was used to generate a pACYCDuet_GGS plasmid lacking the N-terminal His-tag, allowing the His-tag to be used for the purification of mCherry instead. Second, NEBuilder HiFi Assembly was used for subcloning of mCherry-CVLL due to its higher efficiency compared to restriction-based ligation.

First, site-directed mutagenesis was performed using the Q5 Site-Directed Mutagenesis Kit (NEB) to remove the N-terminal 6xHis tag from the GGS gene. Primers (ΔHis_F and ΔHis_R, **Table S2**) were designed using the NEBaseChanger tool. A PCR reaction was set up with 10 ng of template DNA, 500 nM of each primer, and Q5 Hot Start High-Fidelity 2X Master Mix. The following PCR conditions were used: initial denaturation at 98°C for 30 s, followed by 25 cycles of 98°C for 10 s, 63°C for 30 s (annealing), and 72°C for 98 s (extension), with a final extension at 72°C for 2 minutes. After PCR, 1 µL of the product was incubated with the KLD reaction mix for 5 minutes at room temperature to phosphorylate and ligate the amplified DNA and degrade the template DNA via DpnI. Following incubation, 3 µL of the reaction mixture was transformed

into 25 µL of NEB5α competent cells, and colonies were selected using the standard transformation protocol. Positive colonies were confirmed by DNA sequencing.

Next, the modified pACYCDuet_GGS plasmid (1 µg) was linearized by restriction digestion using *NdeI* and *XhoI*. A synthetic gene fragment encoding mCherry fused to an N-terminal 6xHis tag and a C-terminal CVLL peptide was synthesized by Integrated DNA Technologies (IDT). The linearized backbone and synthetic gBlock were combined at a 1:2 molar ratio and incubated with NEBuilder HiFi Master Mix for 30 minutes at 50°C. Following assembly, 2 µL of the reaction mixture was transformed into 25 µL of chemically competent NEB5α cells using the heat shock method, and colonies were selected on LB agar plates supplemented with chloramphenicol (25 µg/mL). Positive colonies were confirmed by DNA sequencing.

For the construction of control pACYCDuet plasmids (lacking GGS), the first step of the procedure was omitted, and the mCherry-CVLL sequence was directly cloned into an unmodified pACYCDuet vector, resulting in the pACYCDuet_mCherry-CVLL plasmid. Additionally, a control plasmid with a mutated AVLL peptide (pACYCDuet_mCherry-AVLL) was constructed by replacing the CVLL sequence with AVLL using Q5 SDM kit using primers (C254A-F and C254A-R, **Table S2**).

2.5 Construction of pET23 vectors

2.5.1 pET23_GGTβ_GGTα

A translationally coupled system was used for the co-expression of the alpha and beta subunits of Rattus norvegicus GGTase-I. The construction of this plasmid has been previously described.[3]

2.5.2 pET23_Control

As a control for 2x2 experiments, a plasmid with a similar backbone but lacking the alpha and beta subunits of Rattus norvegicus GGTase-I was synthesized. The parent plasmid, pET23a_GGTβ_GGTα (1 µg), was linearized by restriction digestion with *XbaI* and *NotI-HF*, which excised the coding regions of the GGT subunits. The digest was separated by agarose gel electrophoresis, and the fragment corresponding to the pET23 backbone was excised and gel-purified. Phosphorylated sense and antisense oligonucleotides (∆GGT-F and ∆GGT-R, **Table S2**) with compatible overhangs were annealed at 95°C for 5 minutes, followed by cooling to room temperature in annealing buffer (50 mM Tris-HCl, 10 mM $MgCl_2$, 1 mM ATP, 10 mM DTT, pH 7.5). The annealed oligonucleotides were ligated into the pET23 backbone using a 1:3 vector-to-insert ratio with a quick ligation kit, incubated at room temperature for 15 minutes. The ligation products were transformed into chemically competent E. coli Eb5α cells using the heat shock method (42°C, 30 seconds) and plated on LB agar containing 150 µg/mL ampicillin. Positive colonies were identified by colony PCR using standard T7 primers and confirmed by DNA sequencing.

3. SDS-PAGE and western blot analysis

SDS-PAGE was conducted using the Laemmli method on a 4–20% gradient Mini-PROTEIN Tris-glycerol (TGX) stain-free SDS-PAGE gel (Bio-Rad). Electrophoresis was performed at 200 V for 30 minutes. For proteins containing tryptophan, the gels were first visualized using the Gel-DOC EZ system with the stain-free protocol. Following visualization, the gels were stained with Simply Blue® (Coomassie-based dye) according to the manufacturer’s protocol and imaged.

For Western blot analysis, proteins were transferred from the SDS-PAGE gel to a 0.2 μm nitrocellulose membrane using a Trans-Blot Turbo Transfer pack (Bio-Rad) at 25V for 7 minutes. The membrane was washed three times with 1X Tris-buffered saline (TBS) and blocked with EveryBlot Blocking Buffer (Bio-Rad) for 1 hour at room temperature with gentle agitation. Membranes were incubated overnight at 4°C in 10 mL of blocking buffer containing 6x-His Tag Monoclonal Antibody (HIS.H8), Alexa Fluor™ 488 (1:2000 dilution). After incubation, the membranes were washed three times for 10 minutes with Tris-buffered saline containing 0.1% Tween 20 (TBS-T) and imaged using ChemiDoc MP imager (Bio-Rad).

4. RP-HPLC

Analytical reverse-phase high-performance liquid chromatography (RP-HPLC) was performed on a Shimadzu LC-2030 instrument equipped with a Phenomenex Jupiter C18 column (5 μm, 300 Å, 250 x 4.6 mm), maintained at room temperature. Detection was carried out using a UV-Vis detector at 190 nm or 210 nm for proteins, and at 214 nm for lipids (in the GGS validation assay). All samples were filtered through a 0.2 μm PVDF filter (Durapore) before analysis. The flow rate was 1 mL/min, and the injection volume was 50 μL.

Due to the differences in solubility and stability of various compounds analyzed in this study (e.g., GGPP vs. geranylgeranylated proteins), different combinations of mobile phases and gradient programs were applied, as described below.

<u>GGS validation</u>

Mobile phase A: 25 mM ammonium bicarbonate (pH 8.0); Mobile phase B (organic): acetonitrile; Gradient: 5-minute isocratic run with 0% B, followed by a linear increase to 90% B at the rate of 2.25%B/min.

<u>Separation of ELP Isoforms</u>

Mobile phase A: water + 0.1% TFA; Mobile phase B: acetonitrile + 0.1% TFA.
Gradient: 5-minute isocratic run with 0% B, followed by a linear increase to 90% B at the rate of 2.25%B/min. After baseline correction, chromatograms were normalized using the following equation:

$$normalization\ factor = \left(\frac{Volume\ of\ cell\ lysate(\mu L)}{Injection\ volume\ (\mu L)}\right) \times \left(\frac{Biomass\ (wet\ pellet, mg)}{Mass\ of\ lysed\ cells\ (mg)}\right)$$

Separation of mCherry Isoforms

Phenomenex Jupiter C4 column (5 µm, 300 Å, 250 x 4.6 mm), Mobile phase A: water + 0.1% TFA; Mobile phase B: acetonitrile + 0.1% TFA. Gradient: 5-minute isocratic run with 0% B, followed by a linear increase to 90% B at the rate of 2.25%B/min. After baseline correction, the chromatograms were normalized using min-max normalization (0-1 range) in GraphPad Prism.

Separation of RhoA and Rap1B Isoforms

Phenomenex Jupiter C18 column (5 µm, 300 Å, 250 x 4.6 mm), Mobile phase A: water + 0.1% TFA; Mobile phase B: acetonitrile + 0.1% TFA. Gradient: 5-minute isocratic run with 0% B, followed by a linear increase to 90% B at the rate of 2.25%B/min. After baseline correction, the chromatograms were normalized using min-max normalization (0-1 range) in GraphPad Prism.

5. MALDI-TOF

MALDI-TOF analysis was performed on a Bruker microflex LP equipped with a micro scout ion source and a nitrogen laser (337 nm), operated in linear positive mode. A solution of sinapinic acid in 70% acetonitrile with 0.1% trifluoroacetic acid (TFA) was used as the matrix. Protein samples were prepared by mixing 5 µL of protein (50 µM in water) with 5 µL of the matrix. Three serial dilutions were performed by a factor of 2 in the matrix solution to obtain a range of protein-to-matrix concentrations. The prepared solutions were applied to a MALDI sample plate and allowed to dry at room temperature.

The instrument was calibrated using dual standards flanking the molecular weight of the protein being analyzed. These standards included Cytochrome C ($[M+H]^+$ = 12,362 Da), Apomyoglobin ($[M+H]^+$ = 16,952.27 Da), and Aldolase ($[M+H]^+$ = 39,212 Da) from the ProteoMass™ Protein MALDI-MS Calibration Kit (Sigma Aldrich). Each MALDI mass spectrum was acquired by averaging 256 laser shots to improve the signal-to-noise ratio. Data were analyzed by Bruker flex control 3.4 software. The theoretical molecular weight and experimentally observed m/z values are reported in **Table S7**.

6. Trypsin digestion and LC-MS

To confirm the selectivity of prenylation to the C-termini CaaX box, trypsin digest followed by LC-MS was employed.

Trypsin digest

All protein samples were digested using a standardized trypsin digestion protocol with minor alterations to account for differences in the solubility of the lipidated peptide fragments. Each sample (50 µg) was reduced with 10 mM TCEP and alkylated with 40 mM chloroacetamide for 30 minutes. Trypsin platinum (Promega) was added at an enzyme-to-protein ratio of approximately 1:50 (w/w), and digestion was allowed to proceed overnight at 37°C. After digestion, samples were acidified with trifluoroacetic acid (TFA, Sigma) and cleaned using stage

tips (3M, 2241).[4] The stage tips were activated with acetonitrile followed by 3% acetonitrile with 0.1% TFA. The eluted peptides were dried, resuspended in a mixture of water, acetonitrile, and formic acid, and analyzed by LC-MS.

Sample-specific alterations:

- Unmodified: No acetonitrile was added to the digest reaction, cleaned using a 3-punch C18 stage tip and eluted with 100 µL of 75% acetonitrile with 0.1% TFA.
- Prenylated (Fr-/GG-modified): Acetonitrile was added to the digest reaction to the final concentration of 20%. Cleaned using a 2-punch MCX stage tip and eluted with 75 µL of 85% acetonitrile with 5% NH_4OH (Sigma).

<u>LC condition</u>

Samples (2 µL) were injected onto a pulled tip nano-LC column with 75 µm inner diameter packed to 25 cm with 3 µm, 120 Å, C18AQ particles (Dr. Maisch). The peptides were separated using a 60 min gradient consisting of mobile phase A: water + 0.1% formic acid and mobile phase B: acetonitrile + 0.1% formic acid.

Gradient program:

Time (min)	Flow (µL/min)	%B
0.00	0.500	3.0
0.01	0.500	3.0
5.00	0.350	5.0
60.00	0.350	85.0
70.00	0.500	85.0
71.00	0.500	3.0
75.00	0.500	3.0

<u>MS condition</u>

The LC column was connected in line with an Orbitrap Lumos via a nanoelectrospray source operating at 2.5 kV. The mass spectrometer was operated using alternating MS1 and targeted MS2 scans. The MS1 scans were collected at 120,000 resolution with a maximum injection time of 50 ms. MS2 scans were performed on the theoretical mass of the lipidated peptide and its oxidized counterpart, in the Orbitrap at 30,000 resolution, following each CID and HCD activation (four MS2 scans per cycle).

7. Protein expression and purification

7.1 Expression and purification of 6xHis-GGS

<u>Expression</u>

The plasmid encoding the N-terminal His-tagged variant of GGS (pET24a_6xHis-GGS) was transformed into *E. coli* BL21(DE3) cells using the heat shock method, followed by selection on LB agar plates containing 90 µg/mL kanamycin. A single colony was inoculated into 50 mL of

2xYT medium with kanamycin (90 µg/mL) and incubated at 37°C with shaking at 200 rpm. After the culture reached an OD600 of 0.7, the cells were harvested by centrifugation (4000 x *g*, 15 min, 4°C), and the pellet was resuspended in 5 mL of PBS buffer. One milliliter of the resuspended pellet was used to inoculate 1 L of 2xYT medium supplemented with kanamycin (90 µg/mL), i.e., 1:100 inoculum. The cultures were incubated at 37°C with shaking (200 rpm), and upon reaching mid-log phase (OD_{600} = 0.7), protein expression was induced with 1 mM IPTG. After 16 h, the cells were harvested by centrifugation (3745 x g, 20 min, 4°C).

<u>Purification</u>

For GGS validation studies, cells were resuspended in lysis buffer and lysed by sonication (3 min, 55–75 Watts, Pulse program: 10 s on, 15 s off). The lysate was clarified by centrifugation (22000 × *g*, 15 min, 4°C), and the His-tagged protein was purified using immobilized metal affinity chromatography (IMAC) on HisPur™ Cobalt Resin (ThermoFisher Scientific) following the manufacturer's protocol, with minor alterations for compatibility with downstream assays as described below.

For *secondary structure determination using circular dichroism (Section 8)*, the bacterial pellet was resuspended in PBS (5 mL per liter of culture). The wash buffer contained 50 mM sodium phosphate, 300 mM NaCl, and 10 mM imidazole (pH 7.2), while the elution buffer contained 50 mM sodium phosphate, 300 mM NaCl, and 150 mM imidazole (pH 7.2). Elution fractions were pooled and dialyzed (MWCO 3 kDa) overnight against 10 mM phosphate buffer (pH 7.4) to remove chloride ion interference during far-UV circular dichroism data collection. Protein concentration in the dialyzed retentate was determined by measuring absorbance at 280 nm, using an extinction coefficient of 55920 $M^{-1}cm^{-1}$ (calculated with ProtParam).

For *in-vitro GGPP synthesis assay (Section 10)*, HEPES-based buffers were used to eliminate the interference of phosphate ions with the BIOMOL® Green assay for pyrophosphate quantification. Cells were lysed in HEPES buffer (100 mM, 137 mM NaCl, pH 7.2). The buffers used for IMAC purification were wash buffer (100 mM HEPES, 300 mM NaCl, 10 mM imidazole, pH 7.2) and elution buffer (100 mM HEPES, 300 mM NaCl, 150 mM imidazole, pH 7.2). Elution fractions were pooled, the protein concentration was determined, and the protein was used for the GGPP synthesis assay.

7.2 Expression and purification of ELP Isoforms

<u>Expression</u>

A pairwise combination of plasmids listed in **Table S3** was used to co-express ELP in the absence or presence of GGS and GGT. A representative protocol for co-expression in the +GGS/+GGT strain (resulting in production of ELP-GG isoforms) is provided below.

The pET23a_GGTβ_GGTα and pACYCDuet_GGS_ELP-CVLL plasmids were co-transformed into E. coli BL21(DE3) competent cells using heat shock (42°C, 30 seconds). Transformed cells were plated on LB agar containing 150 µg/mL ampicillin and 25 µg/mL chloramphenicol for selection. A freshly transformed colony was used to inoculate a 50 mL starter culture of 2xYT medium supplemented with ampicillin (150 µg/mL) and chloramphenicol (25 µg/mL). The starter culture was incubated in an orbital shaker (37°C, 250 rpm) until an OD_{600} of 0.7 was reached.

The cells were harvested by centrifugation (5000 × g, 10 min, 4°C), and the supernatant was discarded. The pellet was resuspended in sterile, chilled PBS (4°C) and used to inoculate 1 L of 2xYT medium supplemented with ampicillin (150 µg/mL) and chloramphenicol (25 µg/mL) at a 1:100 inoculum ratio. The larger cultures were incubated at 37°C with shaking (250 rpm) until the OD_{600} reached 0.7. At this point, the temperature was reduced to 28°C, and 1 mM ZnSO4 (cofactor for GGT, 1000x stock solution) was added to the cultures. After 10 minutes, protein expression was induced by adding IPTG to a final concentration of 0.5 mM. After 16 hours of incubation, the cells were harvested by centrifugation (5000 × g, 35 min, 4°C).

Sample-specific alterations: Hydrophilic ELP isoforms ($ELPA_{40}$) were expressed in T7 Express (NEB cells). A higher concentration of IPTG (1 mM instead of 0.5 mM) was used for protein induction.

Purification

The ELP isoforms were isolated following an established procedure.[5] Briefly, cells were lysed with isopropanol, which resulted in selective partitioning of the ELP into the organic layer. After separating insoluble fractions, the ELPs were precipitated by adding acetonitrile (a non-solvent) to the alcohol layer. The precipitated ELPs were resuspended and purified by RP-HPLC to achieve >95% homogeneity for self-assembly studies. A representative protocol is provided below.

Cell pellets were resuspended in 4 volumes of isopropanol (4 mL/g of wet pellet) by vortexing for 10 minutes, followed by sonication in an ultrasonic bath (VEVOR 15L Ultrasonic, 40 kHz, 25°C). The lysate was centrifuged (15000 x g, 10 minutes, 25°C) to separate the supernatant from the insoluble debris. ELPs were precipitated by adding acetonitrile to the supernatant to achieve a final composition of 70% acetonitrile in isopropanol (v/v), followed by centrifugation (15000 x g, 10 minutes, 4°C). The resulting ELP pellet was resuspended in 50% (v/v) ethanol in water (4 mL/g of ELP pellet) and centrifuged (15000 x g, 5 minutes, 25°C) to remove insoluble impurities. The supernatant was concentrated under reduced pressure (40 °C, 280 mm Hg) to approximately 10 mL, then diluted with deionized water to achieve a 20% ethanol (v/v) solution. The mixture was flash-frozen using liquid nitrogen and subsequently freeze-dried (Labconco FreeZone 4.5 -80°C Benchtop Freeze Dryer).

The lyophilized cake was resuspended in 50% ethanol (v/v), centrifuged (21000 x g, 10 min, 25°C), and the supernatant was filtered (0.22 µm PVDF) before preparative HPLC. Semi-preparative reverse-phase HPLC (RP-HPLC) was performed using a Prominence HPLC system (Shimadzu) with a PDA detector, equipped with a Phenomenex Jupiter® C18 column (5 µm, C18, 300 Å, 250 x 10 mm), at a flow rate of 4.2 mL/min. A mobile phase consisting of a gradient of water (solvent A) and acetonitrile (solvent B), both supplemented with 0.1% TFA, was used to elute the proteins. The gradient consisted of a 5-minute isocratic run at 0% B, followed by a linear increase to 90% B at a rate of 2.25% B/min. Eluted protein fractions were pooled, flash-frozen in liquid nitrogen, and lyophilized. Lyophilized proteins were stored at -20°C.

7.3 Expression and purification of mCherry Isoforms

Expression

A pairwise combination of plasmids listed in **Table S3** was used to co-express mCherry in the absence or presence of GGS and GGT. A representative protocol for co-expression in the +GGS/+GGT strain (resulting in production of mCherry-GG isoform) is provided below.

The pET23a_GGTβ_GGTα and pACYCDuet_GGS_6x-His-mCherry-CVLL plasmids were co-transformed into E. coli BL21(DE3) cells via heat shock and plated on LB agar containing 150 µg/mL ampicillin and 25 µg/mL chloramphenicol. A single colony was inoculated into 50 mL of 2xYT medium with the same antibiotics and incubated at 37°C with shaking (250 rpm) until an OD_{600} of 0.7. The cells were harvested (5000 x g, 10 min, 4°C), resuspended in chilled PBS (4°C), and used to inoculate 1 L of 2xYT medium (1:100 inoculum) supplemented with antibiotics. The culture was incubated at 37°C (250 rpm) until an OD_{600} of 0.7, after which the temperature was lowered to 28°C, and 1 mM ZnSO4 was added. After 10 minutes, protein expression was induced with 1 mM IPTG, and the culture was incubated for 16 hours. The cells were harvested by centrifugation (5000 x g, 35 min, 4°C), resuspended in PBS (5 mL/L of culture), and stored at -80°C before purification.

<u>Purification</u>

Cells were mixed with 4 volumes of bacterial protein extraction reagent (B-PER) supplemented with lysozyme (50 mg/mL), DNase I (2514 U/mL), and 1X Halt protease inhibitor cocktail. After incubating at room temperature for 15 minutes, the lysate was chilled on ice for 5 minutes and then centrifuged at 21000 x g for 20 minutes at 4°C to remove insoluble fractions. The supernatant was supplemented with 10 mM imidazole and incubated with HisPur™ Cobalt Resin (ThermoFisher Scientific) on an end-to-end rotator for 30 minutes at 4°C. After the lysate was removed, the resin was washed three times with 2 volumes of wash buffer (50 mM Tris-HCl, 300 mM NaCl, 40 mM imidazole, pH 7.4). His-tagged proteins were eluted by incubating the resin with 1 volume of elution buffer (50 mM Tris-HCl, 300 mM NaCl, 300 mM imidazole, pH 7.4) for a total of four elution steps. Elution fractions were pooled, concentrated, and buffer-exchanged into PBS using an Amicon Ultra centrifugal concentrator (MWCO 3 kDa). The protein solutions were stored at -80°C.

7.4 Expression and purification of RhoA Isoforms

<u>Expression</u>

The schematic of plasmids used for RhoA expression in +GGS/±GGT strains is shown in Figure S20. A representative protocol for expression in the +GGS/+GGT strain (resulting in the production of the RhoA-GG isoform) is provided below. The control experiment (expression of RhoA in +GGS/-GGT strain) was performed in parallel and under identical conditions, except that ampicillin was omitted, as it was only necessary for maintenance of GGT vector.

The pET23a_GGTβ_GGTα and pACYCDuet_GGS_6xHis-RhoA plasmids were co-transformed into E. coli BL21(DE3) cells via heat shock and plated on LB agar containing 100 µg/mL ampicillin and 25 µg/mL chloramphenicol. A single colony was inoculated into 50 mL of 2xYT medium with the same antibiotics and incubated at 37°C with shaking (250 rpm) until an OD_{600} of 0.7. The cells were harvested (5000 x g, 10 min, 4°C), resuspended in chilled PBS (4°C), and used to inoculate 1 L of 2xYT medium (1:100 inoculum) supplemented with antibiotics. The culture was incubated at 37°C (250 rpm) until an OD_{600} of 0.7, after which the temperature was lowered to 28°C, and 1 mM $ZnSO_4$ was added. After 10 minutes, protein expression was

induced with 1 mM IPTG, and the culture was incubated for 16 hours. The cells were harvested by centrifugation (5000 x g, 35 min, 4°C) and stored at -80°C before purification.

Purification

Cells were lysed with 4 volumes of bacterial protein extraction reagent (B-PER) supplemented with lysozyme (50 mg/mL), DNase I (2514 U/mL), 5mM $MgCl_2$, 5mM β-mercaptoethanol (BME), 10% glycerol, and 1mM phenylmethylsulfonyl fluoride (PMSF). After incubating at room temperature for 15 minutes, the lysate was centrifuged at 21000 x g for 10 minutes at 4°C to remove insoluble fractions. The supernatant was supplemented with wash buffer (25mM Tris, 300 mM NaCl, 5mM $MgCl_2$, 5mM BME, 10% glycerol, 1mM PMSF and 25 mM imidazole, pH 7.4) and incubated with HisPur™ Ni-NTA Resin (Thermo Fisher Scientific) on an end-to-end rotator for 30 minutes at 4°C. After the lysate was removed, the resin was washed three times with 2 volumes of wash buffer. His-tagged proteins were eluted by incubating the resin with 1 volume of native elution buffer (25mM Tris, 300 mM NaCl, 5mM $MgCl_2$, 5mM BME, 10% glycerol, 1mM PMSF and 300 mM Imidazole, pH 7.4) for a total of three elution steps. Elution fractions were analyzed by RP-HPLC.

7.5 Expression and purification of Rap1B Isoforms

Expression

The schematic of plasmids used for Rap1B expression in +GGS/±GGT strains is shown in Figure S21. A representative protocol for expression in the +GGS/+GGT strain (resulting in the production of the Rap1B-GG isoform) is provided below. The control experiment (expression of Rap1B in +GGS/-GGT strain) was performed in parallel and under identical conditions, except that ampicillin was omitted, as it was only necessary for maintenance of GGT vector.

The pET23a_GGTβ_GGTα and pACYCDuet_GGS_6x-His-Rap1B plasmids were co-transformed into E. coli BL21(DE3) cells via heat shock and plated on LB agar containing 100 µg/mL ampicillin and 25 µg/mL chloramphenicol. A single colony was inoculated into 50 mL of 2xYT medium with the same antibiotics and incubated at 37°C with shaking (250 rpm) until an OD_{600} of 0.7. The cells were harvested (5000 x g, 10 min, 4°C), resuspended in chilled PBS (4°C), and used to inoculate 1 L of 2xYT medium (1:100 inoculum) supplemented with antibiotics. The culture was incubated at 37°C (250 rpm) until an OD_{600} of 0.7, after which the temperature was lowered to 28°C, and 1 mM $ZnSO_4$ was added. After 10 minutes, protein expression was induced with 1 mM IPTG, and the culture was incubated for 16 hours. The cells were harvested by centrifugation (5000 x g, 35 min, 4°C) and stored at -80°C before purification.

Purification

Cells were lysed with 4 volumes of bacterial protein extraction reagent (B-PER) supplemented with lysozyme (50 mg/mL), DNase I (2514 U/mL), 5mM $MgCl_2$, 5mM β-mercaptoethanol (BME), 10% glycerol and 1mM phenylmethylsulfonyl fluoride (PMSF). After incubating at room temperature for 15 minutes, the lysate was centrifuged at 21000 x g for 10 minutes at 4°C. After washing the pellet with equal volume of lysis buffer, the inclusion bodies were resuspended in the resolubilization/wash buffer (6M guanidium-HCl, 25mM Tris, 300 mM NaCl, 5mM $MgCl_2$, 5mM BME, 10% glycerol, 1mM PMSF, and 25 mM imidazole, pH 7.4). The solution was briefly sonicated, and then incubated at room temperature on an end-to-end rotator for one hour. After

centrifugation, the resolubilized inclusion bodies were incubated with HisPur™ Ni-NTA Resin (Thermo Fisher Scientific) for 30 minutes at room temperature. The resin was washed three times with 2 volumes of guanidinium wash buffer. His-tagged proteins were eluted by incubating the resin with 1 volume of guanidinium elution buffer (6M guanidium-HCl, 25mM Tris, 300 mM NaCl, 5mM $MgCl_2$, 5mM BME, 10% glycerol, 1mM PMSF and 300 mM Imidazole, pH 7.4) for a total of three elution steps. Elution fractions were analyzed by RP-HPLC.

8. Circular dichroism

Circular dichroism was used to evaluate the secondary structure of recombinantly expressed and purified GGS (Section 7.1) for comparison with MD simulation results. CD spectra were recorded on an AVIV Model 420 CD spectrometer (AVIV Biomedical, Lakewood, NJ, USA). Measurements were performed in the far-UV region (190−250 nm) using a 1.0 mm path length quartz cuvette at 25°C. Each wavelength was scanned for 15 seconds, and the spectra were averaged over three scans. The protein solution was prepared at 5 μM in phosphate buffer (10 mM, pH 7.4). Baseline correction was performed by subtracting the CD spectra of the buffer.

The baseline-corrected CD spectra were converted to mean molar residue ellipticity (MRE) using the following equation:

$$\theta_{MRE} = \frac{\theta}{10 \times C \times Nr \times l}$$

Where

$[\theta]_{MRE}$ = mean molar residue ellipticity ($deg.cm^2.mol^{-1}$)

θ = observed ellipticity in mdeg

C = molar concentration of the protein

Nr = number of residues

l = pathlength in cm

The CD signal was deconvoluted using the BeStSel Server[6] for secondary structure analysis. The results are reported as the mean ± standard deviations of three measurements.

9. Computational modelling

9.1 System building

The crystal structure for *Deinococcus radiodurans* crtE has not been determined yet, but the amino acid sequence is available. We used the AlphaFold 3.0[7] webserver to generate the structure of this protein. The webserver yielded 5 similar models as seen by the small RMSD difference between them (<2 Å). All the models showed the same ranking score of 0.93, which implies a good prediction and hence we selected one of the structures arbitrarily for our usage. The protein structure is shown in **Figure S5a** with chains A and B in different colors.

The protein structure was further validated against other similar synthases with available crystal structures. The multiseq[8] plugin in VMD[9] was used to align a single chain of the following synthases: 1wmw, 1wyo, 2f7m[10], 3oyr[11], and 5djp[12] as shown in **Figure S5b**. The GGS chain A, colored in cyan, was also aligned with the rest of the synthases. The sequence identity of the homologues with respect to the GGS is shown in **Table S1**.

9.2 MD simulation

All molecular dynamics (MD) simulations were performed in NAMD[13] with the CHARMM36[14] force field for the GGS protein, and TIP3P model was used for water. The Gaussian accelerated molecular dynamics (GaMD)[15] used the GPU-offload mode of NAMD 2.14, while the production runs of conventional MD used the GPU resident mode of NAMD 3.0. The temperature was maintained at 310 K by a Langevin thermostat, and the pressure was kept at 1 atm using a Langevin piston barostat. The Particle Mesh Ewald (PME)[16] was used to calculate long-range electrostatic interaction at every time step with a 12 Å cutoff for non-bonded interactions. All bonds involving H-atoms were constrained using the SHAKE[17] algorithm.

Following a 40000-step minimization, a two-step equilibration simulation is performed in which the protein is successively relaxed. The first equilibration, where the GGS was constrained, was run for 100 ps, and the second, where everything was released, was run for 10 ns. Both equilibration steps used a timestep of 1 fs/step. This was followed by a production run of 1.1 μs (2 replicates) with a timestep of 2 fs/step.

9.3 GaMD simulation

Following the equilibration simulation, we also ran GaMD simulations in two steps, boosting only the dihedral energy and not the total potential energy. The first step was a 2ns conventional MD and 50 ns of GaMD equilibration, followed by a 200 ns GaMD production simulation with the upper limit of dihedral boost potential fixed at 15 kcal/mol.

10. GGS validation assay

<u>BIOMOL green assay</u>

Chemically synthesized farnesyl pyrophosphate (FPP) and isopentenyl pyrophosphate (IPP) were obtained from Isoprenoids, LC (United States). Stock solutions of FPP (5 mM) and IPP (10 mM) were prepared by dissolving the compounds in 30% (v/v) ammonium hydroxide in methanol and stored at -80 °C. Recombinant his-tagged *Deinococcus radiodurans* crtE was purified as described in section 7.1, and the concentration of the enzyme was determined by measuring absorbance at 280 nm.

The enzymatic activity of GGS was assessed in vitro using FPP (200 μM) and IPP (366 μM) as substrates. Reactions were carried out in a total volume of 50 μL in an assay buffer composed of 100 mM HEPES (pH 7.5), 5 mM $MgCl_2$, and 10 mM KCl. Recombinant *crtE* was used as the catalyst at a final concentration of 0.5 μM. Control reactions were performed in parallel by omitting one or both substrates (FPP, IPP) or the enzyme (*crtE*), and replacing them with appropriate diluents to maintain a consistent solvent composition across all samples. After the enzyme (or diluent for negative controls) was added, reactions were incubated at 28 °C for 2

hours. To terminate the reaction, 100 µL of BIOMOL GREEN reagent (Enzo Life Sciences) was added to each tube. After 20 minutes, the absorbance at 620 nm was measured using a Nanodrop One spectrophotometer (ThermoFisher Scientific).

Each assay was performed in triplicate, and the results are reported as the mean ± standard deviation. Statistical analysis was conducted using one-way ANOVA, followed by Dunnett's multiple comparison test to compare the positive control reaction (FPP + IPP + *crtE*) to the control groups.

Characterization of reaction products by RP-HPLC and mass spectrometry

To further characterize the reaction products, the enzymatic reactions were scaled up to a total volume of 300 µL, using the same buffer as described above. Following incubation with enzyme, the reaction mixture containing all components (FPP, IPP, and *crtE*) became slightly turbid, hypothesized to be due to the limited solubility of the product (GGPP), in the reaction buffer. Therefore, the reaction mixture was centrifuged (21,000 × *g*, 5 min, RT). After aspiration of the supernatant, the pellet was dissolved in 25 mM ammonium bicarbonate buffer (pH 8), which is a more suitable solvent for GGPP. Both the supernatant and resolubilized pellet fractions were analyzed by RP-HPLC, as described in section 4. GGPP was detected exclusively in the resolubilized pellet fraction, while no significant product was observed in the supernatant (data not shown).

Liquid chromatography-mass spectrometry analysis was conducted to confirm the identity of the reaction products. Reaction mixtures were analyzed using a Thermo Vanquish binary pump LC coupled with a Thermo Quantis triple quadrupole MS. Chromatographic separation was achieved on a Waters XBridge C18 column (3.5 µm, 2.1 x 100 mm). The mobile phase consisted of 25 mM ammonium bicarbonate in water (solvent A) and acetonitrile (solvent B). The gradient program was set as follows:

Time (min)	Flow Rate (mL/min)	%B
0.00	0.300	10
0.20	0.300	10
5.00	0.300	100
5.05	0.600	100
7.00	0.600	100
7.10	0.600	10
9.90	0.600	10
10.00	0.300	10

The MS was operated in negative ion mode using heated electrospray ionization (H-ESI) with a spray voltage of 4500 V. The ion transfer tube temperature was maintained at 300°C, and the vaporizer temperature was 275°C. The scan range was set from 200 to 500 m/z with a resolution (FWHM) of 0.7. Data acquisition and analysis were performed using standard instrument software.

11. VT-Turbidimetry

The temperature-triggered phase-separation behavior of proteins was monitored using a UV-Vis spectrophotometer (Cary 100, Agilent) equipped with a Peltier temperature controller. The absorbance of protein solutions at 350 nm was measured across six concentrations (3.1, 6.2, 12.5, 25, 50, and 100 μM in PBS). The temperature was increased at a rate of 1°C/min from 15°C to a maximum of 65°C, 80°C, or 97°C, depending on the hydrophobicity of the ELP construct. The transition temperature (T_t) at each concentration was defined as the inflection point of turbidimetry plots, corresponding to the maximum of the first derivative of absorbance vs. temperature. Variations in the final temperature did not impact the observed T_t as the phase-separation was complete before reaching these temperatures.

The concentration dependencies of T_t were fitted to two models:

1. **Linear model**: $T_t = T_d - m \times \log([ELP])$, where T_d is the transition temperature of ELP under dilute condition (also known as reference temperature which is a measure of intrinsic hydrophobicity of the sequence) and m represents the concentration dependence of T_t (slope).[18] This empirical model has been shown to predict the behavior of ELPs. The data for both unmodified ELP and GG-modified constructs fit well to this model, as indicated by the R^2 values.
2. **Sigmoidal curve fit**: The concentration dependencies of Fr-modified ELPs across the full concentration range did not fit well to the linear model. Visual inspection suggested a sigmoidal trend, so a four-parameter logistic (4PL) model was used to fit the data:

$$T_t = T_c + \frac{T_d - T_c}{1 + 10^{(\log C_m - \log[ELP]) \times s}}$$

   - **T_c** = Transition temperature at high concentration,
   - **T_d** = Transition temperature at dilute condition
   - **C_m** = concentration at which T_t is halfway between transition temperature at high and low concentrations
   - **s** = (Hill) slope of the curve at C_m.

Each experiment was conducted in triplicate, and the results are reported as mean ± standard deviation. The results are summarized in **Table S9** and **Table S10**.

12. Dynamic light scattering

Dynamic light scattering was performed using a Zetasizer NanoZS (Malvern Panalytical, UK) equipped with a 173° backscattering detector. Protein samples (50 μM in PBS) were prepared at 4°C and filtered into the DLS cuvette using a pre-chilled 0.22 μm PVDF (Durapore) filter. Measurements were conducted across a temperature range of 15–65°C at 1°C increments.

Each sample was equilibrated for 2 minutes at each temperature before recording intensity fluctuations over 11 runs, each lasting 60 seconds.

Autocorrelation functions were analyzed using Zetasizer software (Version 7.11) with the cumulants method to derive the average hydrodynamic diameter (Z-average, Zavg) and polydispersity index (PDI). Intensity distributions were generated using the general-purpose algorithm with default settings. Each DLS run was performed with three technical replicates at each temperature, and the technical replicates were averaged to determine the average hydrodynamic parameters for sample at each temperature. Three results are reported as mean ± standard deviation of three independent samples (**Table S11**)

13. Cryo-TEM

Protein solution (4 µL, 100 µM in PBS) was deposited onto freshly plasma-cleaned Quantifoil grids (Quantifoil Micro Tools GmbH, Germany. The grids were stored in an environmentally controlled chamber at $T < T_t$ for 5 minutes—15 °C for $ELPV_{40}$-GG and 25 °C for both $ELP(V8/A2)_{80}$-GG and $ELPA_{40}$-GG—under 100% humidity. After blotting the excess solution, the grids were vitrified by plunging them into liquid ethane using a Vitrobot Mk IV (Thermo Fisher Scientific) and stored under liquid nitrogen until imaging.

Imaging was performed on a Tecnai BioTwin transmission electron microscope operating at 120 kV, equipped with a Gatan SC1000A CCD camera, and maintained at liquid nitrogen temperature. Images were acquired under low-dose conditions using a Gatan 626 cryo-holder.

For image analysis, ImageJ was used to quantify nanoparticle sizes across multiple images (n=77) for $ELP(V8/A2)_{80}$-GG and (n=73) for $ELPV_{40}$-GG, and histograms of the results were generated using OriginPro. Due to the high hydrophilicity of the $ELPA_{40}$ construct, visualization using cryo-TEM was challenging; however, based on DLS data, we anticipate that these constructs exhibit similar spherical morphologies.

14. Membrane interaction studies

<u>GUV preparation</u>

Giant unilamellar vesicles (GUVs) were prepared using the electroformation method[19] with two distinct lipid compositions. The first lipid mixture consisted of DOPC and NBD-Cholesterol at a weight ratio of 150:1, while the second contained DOPC, DPPC, and NBD-Cholesterol at a weight ratio of 75:75:1. The fluorescent probe NBD-Cholesterol is known to preferentially partition into the disordered regions of lipid membranes, specifically the DOPC-rich domains in mixtures containing both DOPC and DPPC.[20] Both mixtures were dissolved in chloroform at a concentration of 9 mg/mL.

A volume of 10 µL of the lipid mixture was spread on the conductive surface of an indium tin oxide (ITO)-coated glass slide. After overnight evaporation of the solvent, 280 µL of buffer solution (1 mM HEPES, 300 mM glucose, pH = 7.4) was added to the lipid-coated surface within an O-ring chamber, which was then sealed with another ITO-coated slide (with the conductive surfaces facing each other). The electroformation chamber was connected to the Nanion

Vesicle Prep Pro setup (Nanion/Vision-Tek), and GUV formation was induced using a three-step electroformation protocol: (1) the AC voltage was linearly increased from 0 to 3 V peak-to-peak at a frequency of 10 Hz over 15 minutes; (2) the voltage was maintained at 3 V and 10 Hz for 2.5 hours; (3) the frequency was linearly decreased to 4 Hz over 15 minutes. All steps were conducted at 37 °C. The resulting vesicles were collected, diluted 1:4 in the storage buffer (10 mM HEPES, 150 mM NaCl, pH= 7.4), and stored at room temperature for use within 24 hours.

GUV-binding assays

GUV-binding assays were performed in a μ-Slide 8 Well glass chamber (ibidi). The glass surface was passivated by incubating with 200 μL of 2 mg/mL BSA for 1 hour at room temperature. After incubation, the chamber was rinsed with distilled water (3 rinses, 200 μL each) to remove unbound BSA. Next, 100 μL of the GUV solution was added to the wells, followed by the addition of protein solutions to achieve a final protein concentration of 1 μM.

After a 15-minute incubation, the mixtures were imaged using a Zeiss LSM 980 Airyscan 2 confocal microscope equipped with a 63× oil immersion objective (NA 1.4). NBD-Cholesterol-containing GUVs were excited at 488 nm, and emission was detected from 491 to 585 nm. mCherry was excited at 561 nm, and emission was detected from 591 to 705 nm.

15. Supplementary tables

Table S1. Sequence identity of selected synthase homologues of *D. radiodurans* CrtE

PDB ID	Name	Sequence identity
1wmw	Geranylgeranyl diphosphate synthetase	51.5%
1wyo	Geranylgeranyl pyrophosphate synthetase	30.3%
2f7m	Farnesyl pyrophosphate synthase	26.5%
3oyr	Trans-isoprenyl diphosphate synthase	29.6%
5djp	Farnesyl diphosphate synthase	26.5%

Table S2. Sequence of oligonucleotides used in this study

Name	Sequence (5′-3′)
GGS-F	GTT TAA CTT TAA TAA GGA GAT ATA CAT GCA TCA TCA TCA CCA CCA CCG CCC G
GGS-R	AGC TTG TCG ACC TGC AGG TCA TTA TCA TTT TTC GCG GGT GGC
ΔGGT-F	CTA GTC TTG GGC TTC AAC GA
ΔGGT-R	GGC CTC GTT GAA GCC CAA GA
ΔHis-F	CGC CCG GAA TTA CTC
ΔHis-R	CGC CCG GAA TTA CTC
C254A-F	ATA TAA GGG CGC GGT TCT TTT GTA ATG ATA AG
C254A-R	AATTCGTCCATACCCC

Table S3. Plasmids used in this study[a]

Plasmid name	Features
pET24a_GGS	Kan^r, monocistronic T7 promotor
pET24a_ELP-CVLL	Kan^r, monocistronic T7 promotor
pACYCDuet_()_()	Cmp^r, p15A Ori, bicistronic T7 promotor, MCS 1=empty, MCS2= empty
pACYCDuet_GGS_()	Cmp^r, p15A Ori, bicistronic T7 promotor, MCS 1=GGS, MCS2= empty
pACYCDuet_()_POI-CVLL	Cmp^r, p15A Ori, bicistronic T7 promotor, MCS 1=empty, MCS2= POI-CVLL
pACYCDuet_GGS_POI-CVLL	Cmp^r, p15A Ori, bicistronic T7 promotor, MCS 1=GGS, MCS2= POI_CVLL
pET23_GGT	Amp^r, pBR322 Ori, monocistronic T7 promotor, translation coupling of GGTβ and GGTα subunits
pET23_()	Amp^r, pBR322 Ori, monocistronic T7 promotor

[a] POI refer to $ELPV_{40}$, $ELP(V8/A2)_{80}$, $ELPA_{40}$, and mCherry. An N-terminal His-tagged GGS was used for co-expression with ELP constructs.

Table S4. Strains used in this study[a]

Strains	GGS	GGT	Plasmid combination
1	+	+	pET23a_GGT + pACYCDuet_GGS_POI-CVLL
2	-	+	pET23a_GGT + pACYCDuet_()_POI-CVLL
3	+	-	pET23_() + pACYCDuet_GGS_POI-CVLL
4	-	-	pET23_() + pACYCDuet_()_POI-CVLL

[a] Strains are derived from co-transformation of the indicated plasmids to BL21(DE3) or T7 Express cells.

Table S5. Summary of fragments and assignments from LC-MS/MS analysis of prenylated peptides

Ion	GC$_{Fr}$VLL			GC$_{GG}$VLL		
	Calculated m/z	Observed m/z		Calculated m/z	Observed m/z	
		CID	HCD		CID	HCD
$[M+H]^+$	708.4728	708.4731	708.4731	776.5354	776.5375	776.5375
MH-H_2O	690.4623	690.4617	n.d.[a]	758.5249	758.5240	n.d.
MH-prenyl-H_2O	486.2745	486.2744	n.d.	486.2649	486.2740	n.d.
a4	549.3800	549.3831	n.d.	617.4500	617.4454	n.d.
a3	436.3000	436.2991	436.2986	504.3600	504.3608	n.d.
b4	577.3774	577.3778	n.d.	645.4400	645.4404	n.d.
b3	464.2933	464.2939	464.2935	532.3567	532.3564	532.3557
b2	365.2249	365.2256	365.2254	433.2883	433.2878	433.2876
b4* (b4-prenyl)	373.1900	373.1904	373.1902	373.1900	373.1902	373.1901
b3* (b3-prenyl)	260.1100	260.1063	260.1061	260.1100	260.1060	260.1060
b2* (b2-prenyl)	161.0400	n.d.	161.0378	161.0400	n.d.	161.0376
b3** (b3- thioprenyl)	227.1300	226.1185	226.1183	227.1300	226.1181	226.1182
y3	344.2544	344.2544	344.2544	344.2544	344.2543	n.d.
y2	245.1860	245.1859	245.1858	245.1860	n.d.	245.1855
y1	132.1019	n.d.	132.1018	132.1019	n.d.	133.0428

[a] n.d. no peak was observed

Table S6. Sequence of proteins used in this study

Protein Name	Sequence
GGTα	(M)AATEGVGESAPGGEPGQPEQPPPPPPPPPAQQPQEEEMAAEAGEAAASPMDDGFLSLDSP<br>TYVLYRDRAEWADIDPVPQNDGPSPVVQIIYSEKFRDVYDYFRAVLQRDERSERAFKLTRDAIELN<br>AANYTVWHFRRVLLRSLQKDLQEEMNYITAIIEEQPKNYQVWHHRRVLVEWLKDPSQELEFIADIL<br>NQDAKNYHAWQHRQWVIQEFRLWDNELQYVDQLLKEDVRNNSVWNQRHFVISNTTGYSDRAV<br>LEREVQYTLEMIKLVPHNESAWNYLKGILQDRGLSRYPNLLNQLLDLQPSHSSPYLIAFLVDIYED<br>MLENQCDNKEDILNKALELCEILAKEKDTIRKEYWRYIGRSLQSKHSRESDIPASV
GGTβ	(M)AATEDDRLAGSGEGERLDFLRDRHVRFFQRCLQVLPERYSSLETSRLTIAFFALSGLDMLDSL<br>DVVNKDDIIEWIYSLQVLPTEDRSNLDRCGFRGSSYLGIPFNPSKNPGTAHPYDSGHIAMTYTGLS<br>CLIILGDDLSRVDKEACLAGLRALQLEDGSFCAVPEGSENDMRFVYCASCICYMLNNWSGMDMK<br>KAISYIRRSMSYDNGLAQGAGLESHGGSTFCGIASLCLMGKLEEVFSEKELNRIKRWCIMRQQNG<br>YHGRPNKPVDTCYSFWVGATLKLLKIFQYTNFEKNRNYILSTQDRLVGGFAKWPDSHPDALHAYF<br>GICGLSLMEESGICKVHPALNVSTRTSERLRDLHQSWKTKDSKQCSDNVHISSEEF
GGS	(M)HHHHHHRPELLARVLSLLPETSATPELARFYALLRDYPQRGGKGIRSELLLASARAHGLSESD<br>TGWESALWLAAALELFQNWVLVHDDIEDDSEERRGRPALHHLCGMPVALNVGDALHAYMWAAV<br>GKANVPGAFEEFLQMVYRTAEGQHLDLAWVEGREWGLRPADYLQMVGLKTAHYTVIVPLRLGAL<br>AAGMAPQDAFTPAGLALGTAFQIRDDVLNLAGDPVKYGKEIGGDLLEGKRTLIVLDWLTTAPDDR<br>KAIFLDQMRRHRADKDPAVIDEIHRWLLESGSVEAAQDYAQAQAAEGLDLLEKALADAPDAQAAA<br>ALLASVRELATREK
ELPV$_{40}$-CVLL	(M)GHHHHHHHHGVGVPGVGVPGVGVPGVGVPGVGVPGVGVPGVGVPGVGVPGVGVPGVGV<br>PGVGVPGVGVPGVGVPGVGVPGVGVPGVGVPGVGVPGVGVPGVGVPGVGVPGVGVPGVGV<br>PGVGVPGVGVPGVGVPGVGVPGVGVPGVGVPGVGVPGVGVPGVGVPGVGVPGVGVPGVGV<br>PGVGVPGVGVPGVGVPGVGVPGVGVPGVGVPGCVLL
ELP(V8/A2)$_{80}$ -CVLL	(M)GVGVPGVGVPGAGVPGVGVPGVGVPGVGVPGVGVPGAGVPGVGVPGVGVPGGKGVGVP<br>GVGVPGAGVPGVGVPGVGVPGVGVPGVGVPGAGVPGVGVPGVGVPGGKGVGVPGVGVPGA<br>GVPGVGVPGVGVPGVGVPGVGVPGAGVPGVGVPGVGVPGGKGVGVPGVGVPGAGVPGVGV<br>PGVGVPGVGVPGVGVPGAGVPGVGVPGVGVPGGKGVGVPGVGVPGAGVPGVGVPGVGVPG<br>VGVPGVGVPGAGVPGVGVPGVGVPGGKGVGVPGVGVPGAGVPGVGVPGVGVPGVGVPGVG<br>VPGAGVPGVGVPGVGVPGGKGVGVPGVGVPGAGVPGVGVPGVGVPGVGVPGVGVPGAGVP<br>GVGVPGVGVPGGKGVGVPGVGVPGAGVPGVGVPGVGVPGVGVPGVGVPGAGVPGVGVPGV<br>GVPGGKGCVLL
ELPA$_{40}$-CVLL	(M)SKGPGAGVPGAGVPGAGVPGAGVPGAGVPGAGVPGAGVPGAGVPGAGVPGAGVPGAGV<br>PGAGVPGAGVPGAGVPGAGVPGAGVPGAGVPGAGVPGAGVPGAGVPGAGVPGAGVPGAGV<br>PGAGVPGAGVPGAGVPGAGVPGAGVPGAGVPGAGVPGAGVPGAGVPGAGVPGAGVPGAGV<br>PGAGVPGAGVPGAGVPGAGVPGAGVPGCVLL
mCherry-CVLL	(M)HHHHHHGVSKGEEDNMAIIKEFMRFKVHMEGSVNGHEFEIEGEGEGRPYEGTQTAKLKVTK<br>GGPLPFAWDILSPQFMYGSKAYVKHPADIPDYLKLSFPEGFKWERVMNFEDGGVVTVTQDSSLQ<br>DGEFIYKVKLRGTNFPSDGPVMQKKTMGWEASSERMYPEDGALKGEIKQRLKLKDGGHYDAEV<br>KTTYKAKKPVQLPGAYNVNIKLDITSHNEDYTIVEQYERAEGRHSTGGMDELYKGCVLL
RhoA	(M)GSSHHHHHHAAIRKKLVIVGDGACGKTCLLIVFSKDQFPEVYVPTVFENYVADIEVDGKQVEL<br>ALWDTAGQEDYDRLRPLSYPDTDVILMCFSIDSPDSLENIPEKWTPEVKHFCPNVPIILVGNKKDL<br>RNDEHTRRELAKMKQEPVKPEEGRDMANRIGAFGYMECSAKTKDGVREVFEMATRAALQARR<br>GKKKSGCLVL
Rap1B	(M)GSSHHHHHHREYKLVVLGSGGVGKSALTVQFVQGIFVEKYDPTIEDSYRKQVEVDAQQCML<br>EILDTAGTEQFTAMRDLYMKNGQGFALVYSITAQSTFNDLQDLREQILRVKDTDDVPMILVGNKCD<br>LEDERVVGKEQGQNLARQWNNCAFLESSAKSKINVNEIFYDLVRQINRKTPVPGKARKKSSCQLL

Table S7. Summary of mass spectrometry characterization of constructs using MALDI-TOF (full length proteins)

Constructs	Theoretical		Observed m/z
	Monoisotopic (Da)	Average (Da)	
ELPV$_{40}$-GG	18298.32	18309.74	18298
ELPV$_{40}$-Fr	18230.26	18241.62	18241
ELPV$_{40}$-CVLL	18026.07	18037.27	18027
ELP(V8/A2)$_{80}$-GG	35002.73	35024.35	35013
ELP(V8/A2)$_{80}$-Fr	34934.67	34956.23	34954
ELP(V8/A2)$_{80}$-CVLL	34730.48	34751.88	34742
ELPA$_{40}$-GG	16392.78	16402.82	16393
ELPA$_{40}$-Fr	16324.72	16334.70	16323
ELPA$_{40}$-CVLL	16120.53	16130.35	16122
His-mCherry-GG[a]	28322.05	28340.16	28348
His-mCherry-Fr[a]	28253.99	28272.04	28274
His-mCherry-CVLL[a]	28049.8	28067.69	28069

a. The theoretical mass includes the initial methionine, with a -20 Da adjustment to account for fluorophore formation

Table S8. Summary of mass-spectrometry characterization of trypsinized C-terminal fragments [a]

Constructs	Theoretical monoisotopic mass for $[M+H]^+$ (Da)	Observed m/z	Δ (%)
$GC_{GG}VLL$	776.5354	776.5377	0.0003
$GC_{GG}(O)VLL$	792.5303	792.5308	0.0001
$GC_{Fr}VLL$	708.4728	708.4731	0.00004
$GC_{Fr}(O)VLL$	724.4677	724.4685	0.0001
$GC_{(CAM)}VLL$	561.3064	561.3057	-0.0001
$SSC_{GG}QLL$	922.5682	922.5698	0.0001
$SSC_{GG}(O)QLL$	938.5631	938.5647	0.0001
$SSC_{(CAM)}QLL$	707.3393	707.3398	0.0001
$SGC_{GG}LVL$	863.5675	863.5689	0.0001
$SGC_{GG}(O)LVL$	879.5624	879.5640	0.0001
$SGC_{(CAM)}LVL$	648.3387	648.3385	-0.00003

[a] GG = Geranylgeranyl; Fr = Farnesyl; CAM = carboxymethylamide

Table S9. Concentration-dependent transition temperature of ELP isoforms[a]

[Protein] (µM)	Transition temperature (T_t ˚C)								
	ELPV$_{40}$			ELP(V8/A2)$_{80}$			ELPA$_{40}$		
	-CVLL	-Fr	-GG	-CVLL	-Fr	-GG	-CVLL	-Fr	-GG
100	30.7±1.9	17.7±1.3	17.9±0.3	43.8±0.3	31.0±0.1	31.6±0.7	91.0±0.4	66.9 ±0.9	61.4±1.8
50	32.0±2.1	17.9±1.1	17.9±0.5	45.6±0.6	31.7±0.1	31.7± 0.1	95.9±0.0	85.9±1.2	62.6±1.7
25	33.1±2.4	20.6±1.9	18.1± 0.6	47.7±0.1	33.7±1.2	32.0± 0.4	n.d.[b]	n.d.	64.1±1.4
12.5	34.9±3.6	25.6±1.3	19.3±0.4	49.4±0.6	37.0±1.2	32.2± 0.8	n.d.	n.d.	66.4±1.2
6.2	35.0±0.5	25.8±3.0	19.4±0.7	52.0±0.7	39.9±1.3	33.0± 0.6	n.d.	n.d.	67.6±0.3
3.1	44.0±3.2	28.8±2.0	20.8±2.0	54.8±0.1	43.7±1.5	34.1± 0.7	n.d.	n.d.	n.d.

[a] mean ± SD (n=3);
[b] n.d. no transition temperature was observed.

Table S10. Fitted parameters quantifying the concentration-dependent variation of transition temperatures for ELP constructs[a]

Construct	Linear Regression	
	m ([˚C / Log (µM/µM)])	T_d (˚C)
ELPV$_{40}$-CVLL	- 7.4	44.2
ELPV$_{40}$-GG	-1.9	21.3
ELP(V8/A2)$_{80}$-CVLL	-7.6	58.5
ELP(V8/A2)$_{80}$-GG	-2.2	35.1
ELPA$_{40}$-CVLL	n.a.[b]	n.a.
ELPA$_{40}$-GG	-5.4	72.0

Construct	Sigmoidal Fit			
	T_c (˚C)	T_d (˚C)	C_m (µM)	*s*
ELPV$_{40}$-Fr	17.2	28.0	18.6	-2.3
ELP(V8/A2)$_{80}$-Fr	30.7	48.4	6.9	-1.2
ELPA$_{40}$-Fr	n.a.	n.a.	n.a.	n.a.

[a] Refer to section 11 for definitions of each parameter.
[b] n.a. Data was not fitted because transition temperatures for concentrations below 50 µM exceeded 95°C, which was outside the experimental range.

Table S11. Summary of VT-DLS results for GG-modified ELPs

ELPV40-GG			ELP(V8/A2)80-GG			ELPA40-GG		
T (˚C)	Z_{avg} (d.nm)	PDI	T (˚C)	Z_{avg} (d.nm)	PDI	T (˚C)	Z_{avg} (d.nm)	PDI
13	33±6	0.2±0.1	24	36±2	0.1±0	15	44±3	0.2±0.1
14	33±6	0.2±0.2	25	36±2	0.1±0	25	41±4	0.2±0.1
15	34±7	0.2±0.2	26	36±3	0.1±0.1	35	40±0.8	0.2±0.1
16	35±8	0.2±0.1	27	37±4	0.1±0.1	45	39±3	0.3±0.2
17	43±9	0.5±0.2	28	38±5	0.1±0.1	50	39±0.9	0.3±0.2
18	1422±1318	0.9±0.1	29	39±6	0.1±0.1	51	38±1	0.3±0.2
19	6153±1436	0.8±0.2	30	39± 5	0.1±0.1	52	37±3	0.3±0.2
20	9455±3882	0.8±0.2	31	1577±2177	0.5±0.7	53	40±2	0.3±0.2
21	9145±3752	0.8±0.2	32	11898±3962	1±0.0	54	39±2	0.3±0.2
22	10160±4565	0.7±0.2	33	18100±2616	1±0.0	55	39±2	0.3±0.2
23	10352±4298	0.8±0.2	34	26090±6109	0.8±0.2	56	39±3	0.3±0.2
24	11034±4654	0.8±0.2	35	25665±940	0.9±0.0	57	38±2	0.3±0.2
25	11784±5812	0.8±0.2	36	30345±898	0.8±0.0	58	39±3	0.3±0.2
						59	43±4	0.2±0.2
						60	1160±276	0.4±0.1
						61	2190±210	0.8±0.4
						62	2946±225	0.8±0.4
						63	3503±298	0.7±0.5
						64	3977±448	0.8±0.3
						65	4410±568	0.6±0.4
						66	4722±206	0.6±0.4

Z_{avg} = Hydrodynamic diameter and PDI data represent mean ± SD (n = 3).

16. Supplementary figures

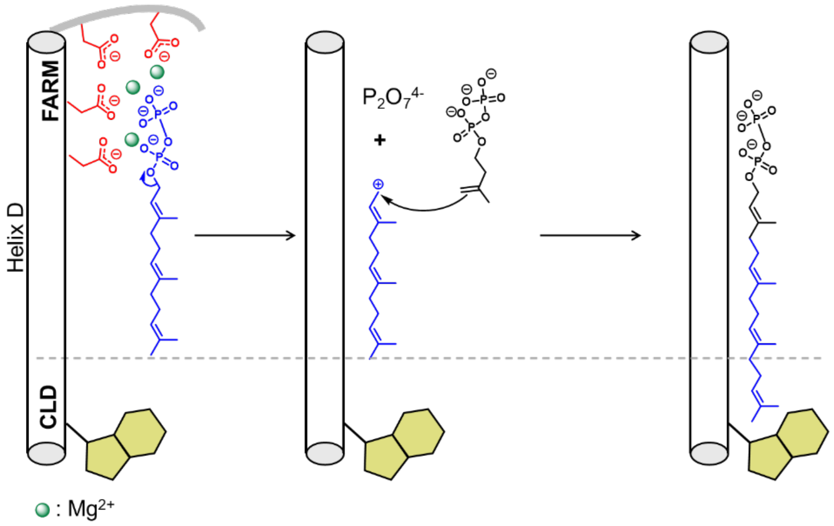

Figure S1. Schematic of the chain elongation reaction catalyzed by medium-chain (E)-prenyl diphosphate synthases. The schematic illustrates the stepwise addition of isopentenyl diphosphate (IPP, black) to farnesyl pyrophosphate (FPP, blue), resulting in the elongation of the chain by one isoprene unit per step, with geranylgeranyl pyrophosphate (GGPP) as the final product. FARM: First aspartic-rich motif; CLD: Chain-length determining region.

Supplementary Note 1. Liu et al. identified a putative GGS enzyme in *D. radiodurans* through homology and genome mining.[21] This protein shares high homology with the prenyl synthase family, featuring two conserved aspartic acid-rich motifs for coordinating Mg^{2+} ions that position the diphosphate of the allylic substrate (e.g., FPP), and promote the cleavage of its carbon–oxygen bond to form a carbocation. The carbocation then reacts with IPP, leading to elongation of the prenyl chain (**Figure S1**). This elongation process continues with sequential IPP additions, and enzymes in this family are named after their final product (e.g., farnesyl diphosphate synthase).

Although the mechanism for determining the product chain length for these enzymes is known, there is no simple relationship between function and primary sequence, making it challenging to assign such functions a priori.[11] For instance, the sequence of *D. radiodurans* GGS shares motifs with both eukaryotic GGPPS (DDXXDD motif) and prokaryotic enzymes (hydrophobic aromatic residues upstream of FARM), which typically favor shorter prenyl chains like FPP due to steric hindrance (**Figure 1a**). Due to these mixed features and a lack of direct characterization, we prioritized in vitro studies to confirm the enzyme's ability to synthesize GGPP from *E. coli*'s endogenous FPP and IPP.

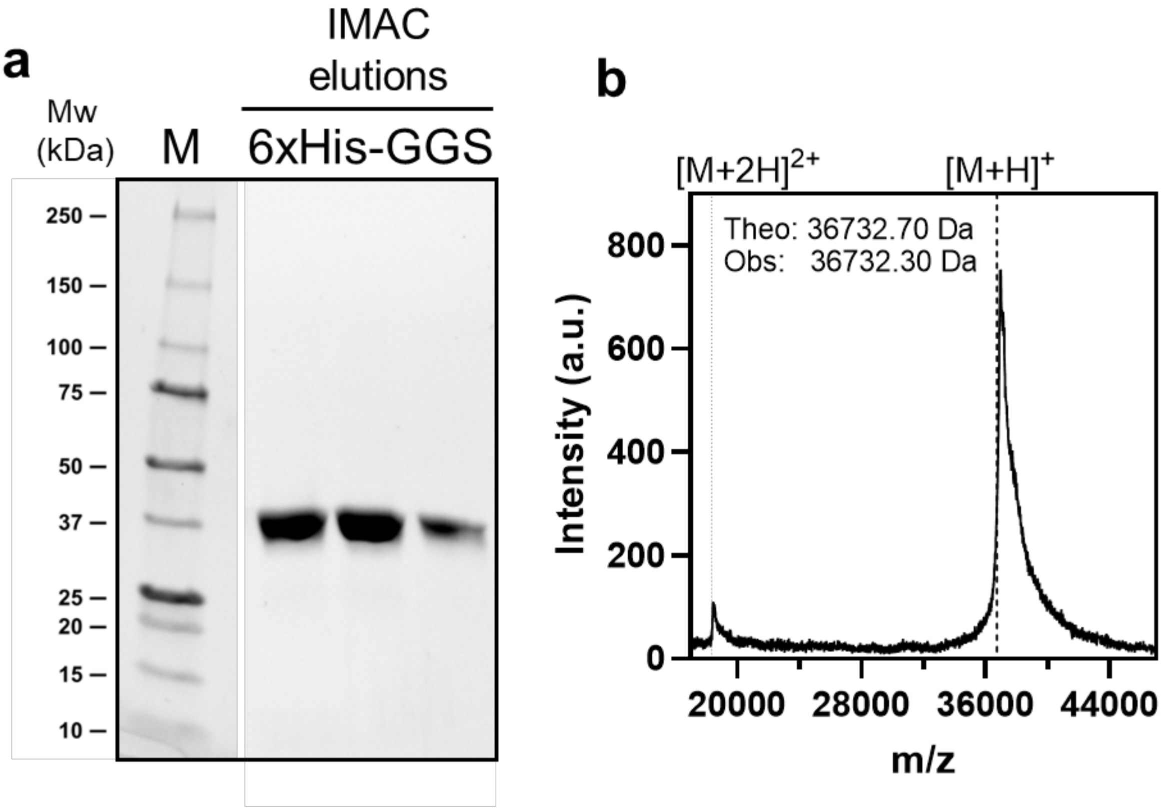

Figure S2. Characterization of recombinantly expressed N-terminal His-tagged putative GGS. (a) SDS-PAGE analysis of elutions following IMAC purification, visualized using stain-free technology, confirming the presence of the purified protein. **(b)** MALDI-TOF-MS of purified proteins, with dotted and dashed lines indicating $[M+2H]^{2+}$ and $[M+H]^{+}$ molecular ion peaks, respectively.

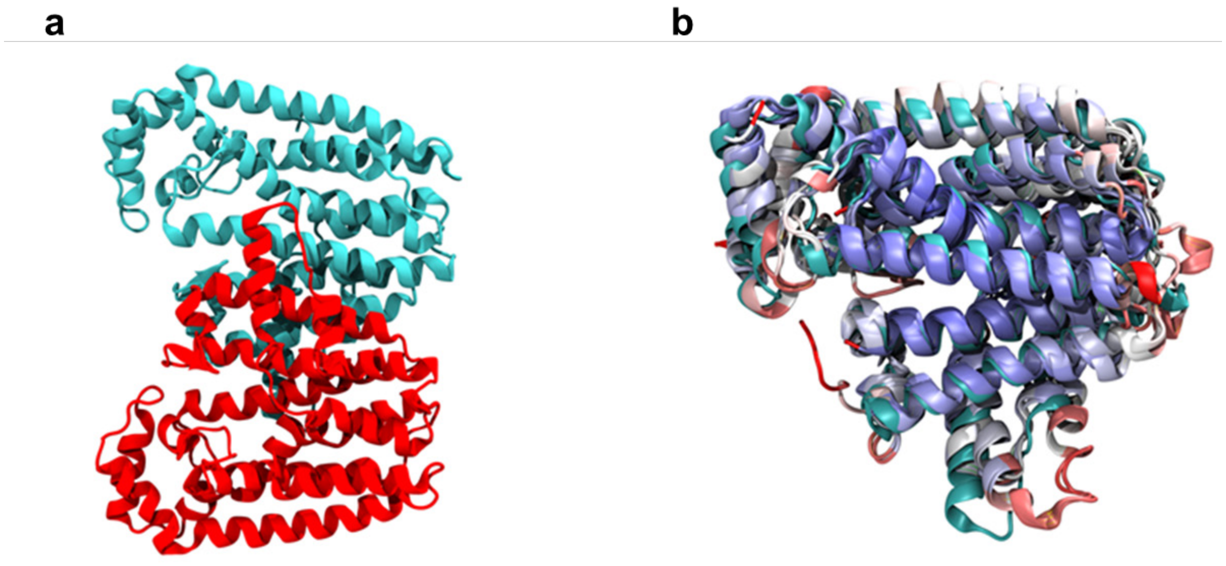

Figure S3. Simulated quaternary structure of *D. radiodurans* GGS and its alignment with homologous synthases. (a) Homodimeric structure of GGS with chain A shown in cyan and chain B in red. **(b)** Aligned structures of GGS and homologous synthases (**Table S1**), colored according to their Qres values as measured by the MultiSeq plugin. The GGS chain is highlighted in cyan to clearly demonstrate the structural overlap with the homologous enzymes. Qres or the “Q” value per residue is a measure of structural homology. A higher Q value (bluish hue) implies strong structural overlap, and a low Q value (reddish hue) means structures are not aligned well.

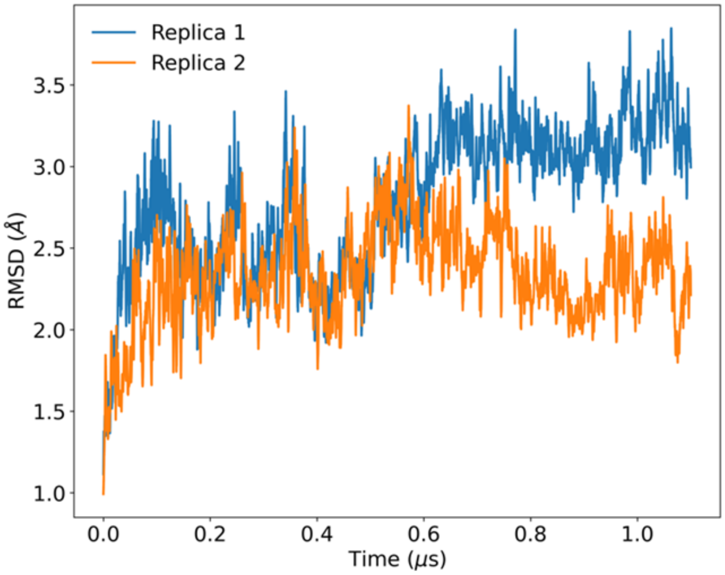

Figure S4. **The root mean square deviation (RMSD) of the two GGS production runs**. A 10-point rolling average is applied to smooth fluctuations over production time (1.1 μs).

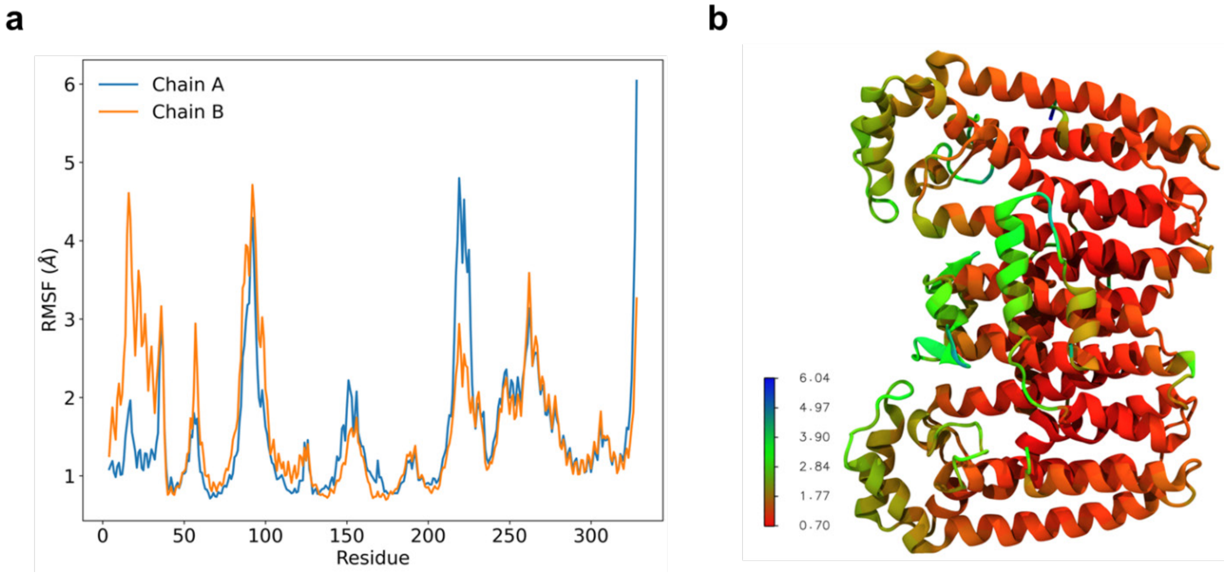

Figure S5. (a) RMSF plot for chain A and B of the GGS during the production simulation. **(b)** The RMSF projected on the structure of the protein.

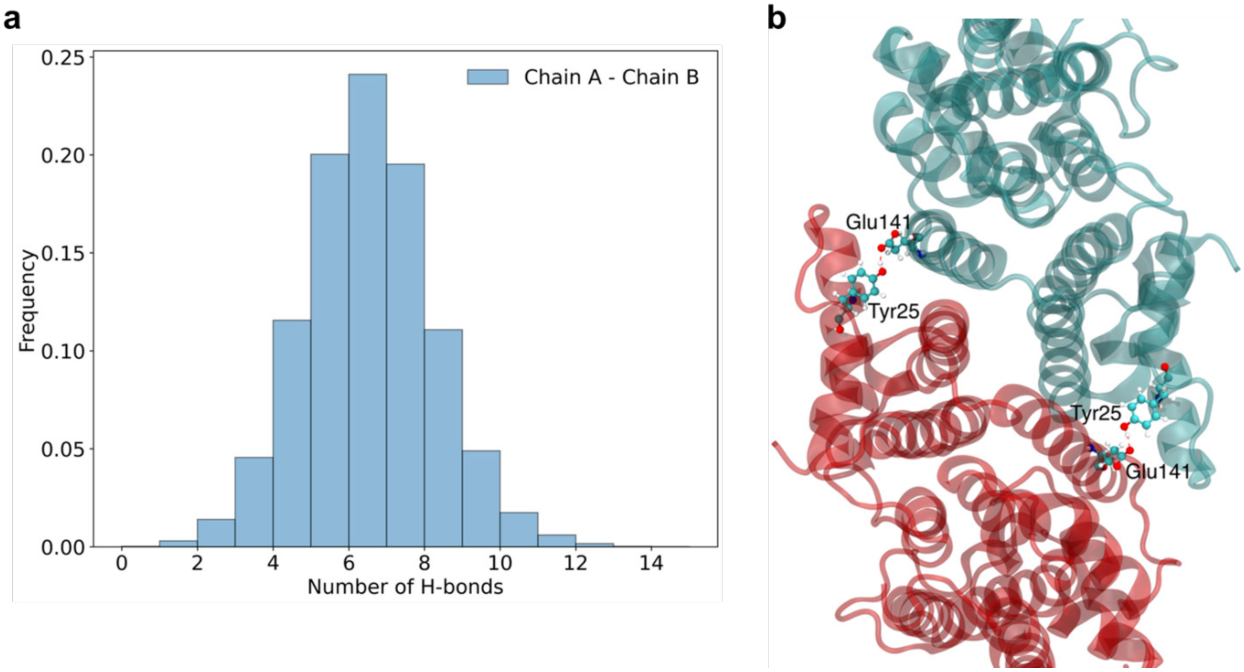

Figure S6. (a) Histogram of hydrogen bonds for the GGS production run. **(b)** H-bond between Tyr25 and Glu141 in both chains. Chain A is represented in cyan and chain B is red.

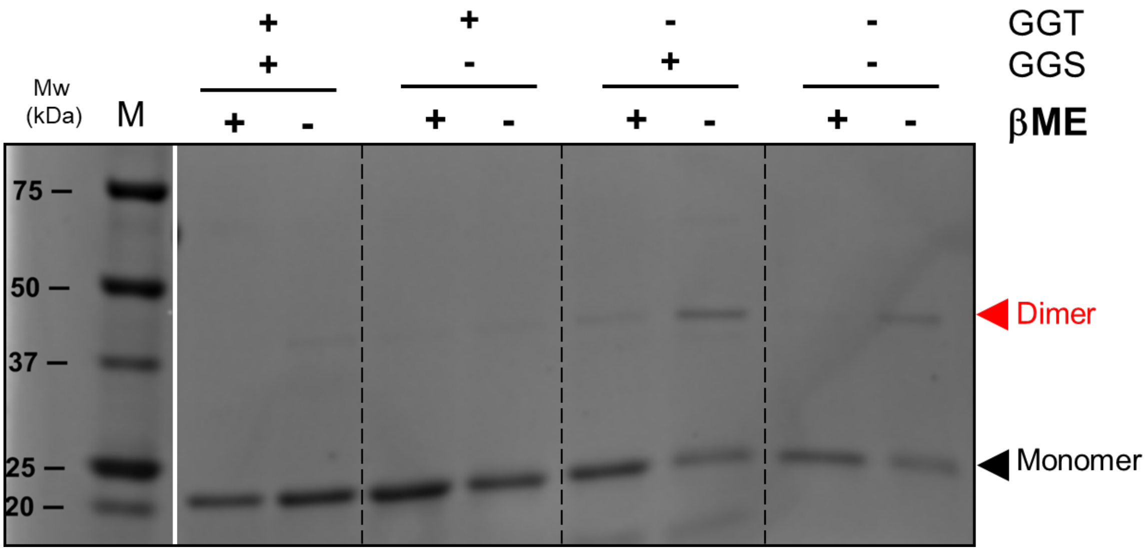

Figure S7. SDS-PAGE analysis of protein isoforms obtained from expression of a model ELP in (±GGS/±GGT) strains. Consistent with HPLC results, a significant amount of ELP dimers (apparent Mw ~ 40 kDa) are observed in -GGT strains. The addition of beta-mercaptoethanol (βME) to the gel loading buffer leads to the disappearance of these bands, indicating disulfide bond formation between the chains.

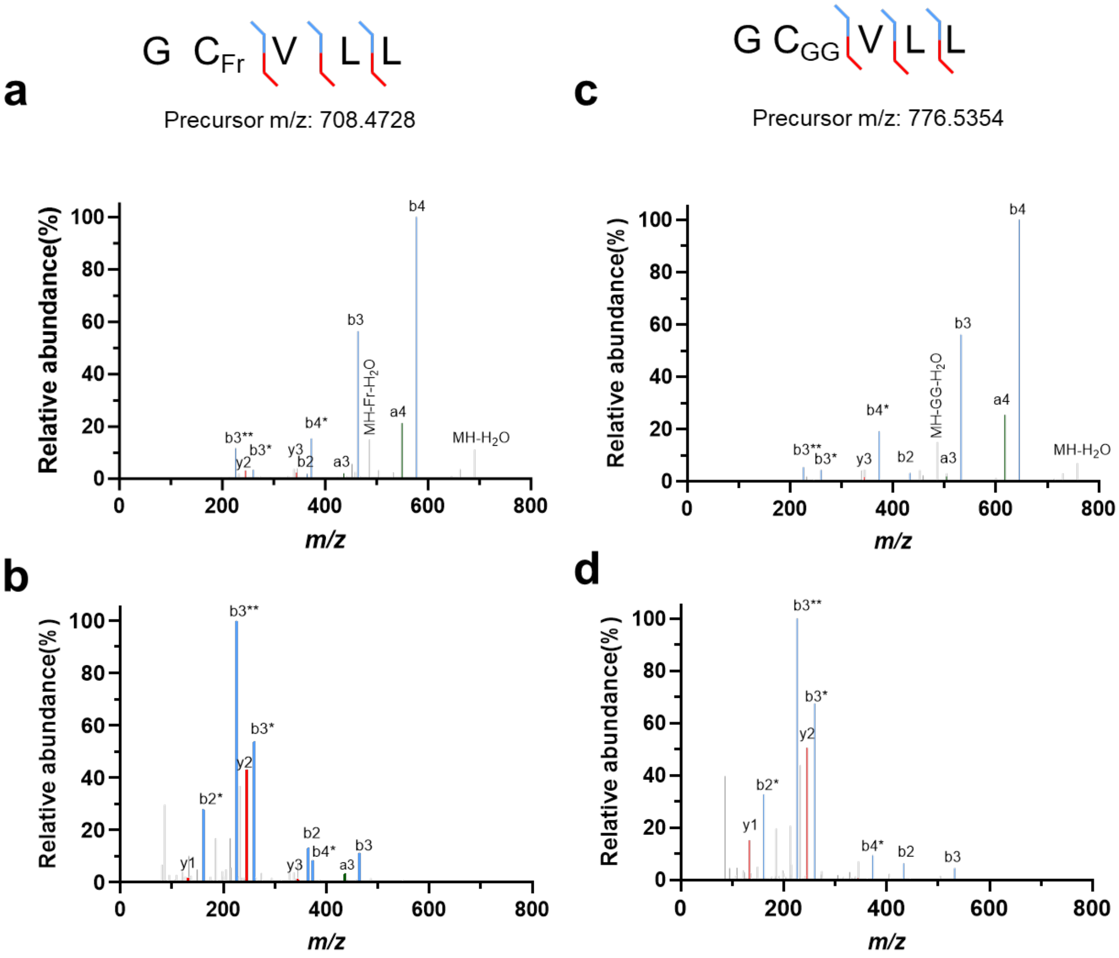

Figure S8. MS/MS analysis of prenylated C-terminal peptide fragments. (a) MS/MS spectrum of Fr-modified peptide fragment obtained using collision-induced dissociation (CID); **(b)** MS/MS spectrum of Fr-modified peptide fragment using higher-energy collisional dissociation (HCD); **(c)** MS/MS spectrum of GG-modified peptide fragment using CID; **(d)** MS/MS spectrum of GG-modified peptide fragment using HCD.

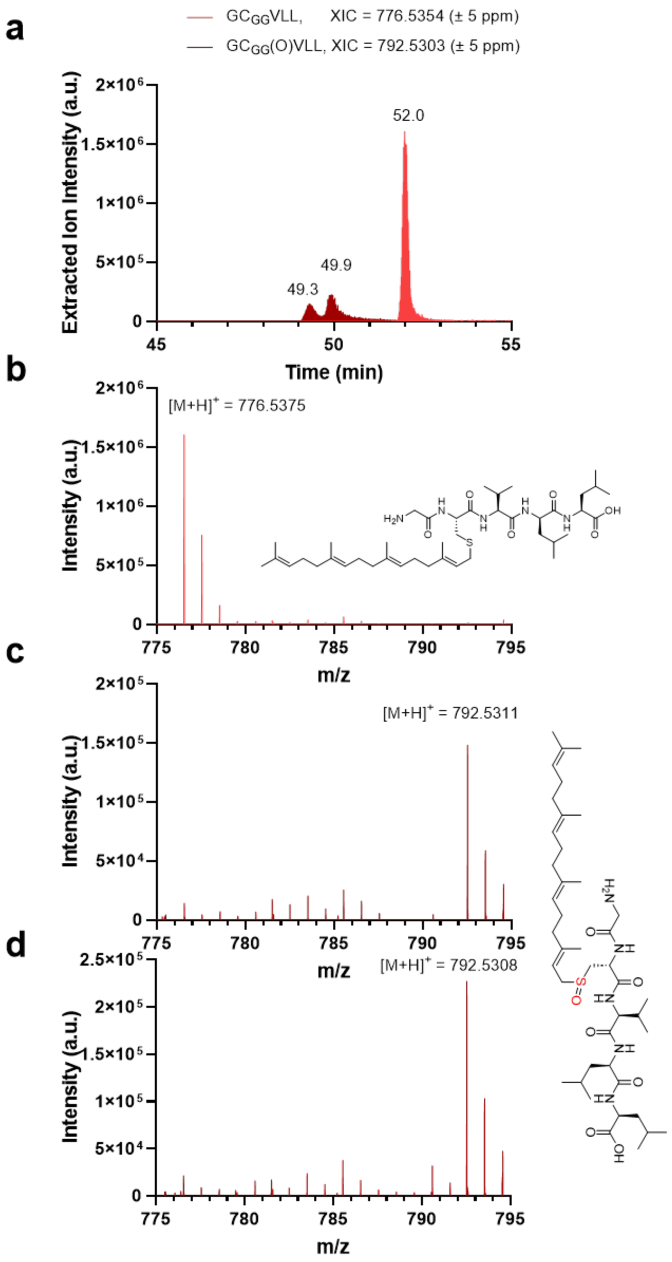

Figure S9. LC-MS analysis of the reduced and oxidized forms of the lipidated C-terminal peptide fragment. (a) Extracted Ion Chromatogram (XIC) of trypsin-digested ELP(V8/A2)$_{80}$ expressed in (+GGS/+GGT) strains shows a major peak at 52 min (red trace), corresponding to the geranylgeranylated GCVLL peptide, and two minor peaks at 49.3 and 49.9 min (maroon trace), which correspond to the oxidized (sulfoxide) form of the GG-modified peptide. (b) MS spectrum of the 52-min peak confirms the expected mass of the geranylgeranylated peptide. (c, d) MS spectra of the earlier-eluting peaks at 49.3 and 49.9 min reveal a +16 Da mass shift, consistent with sulfoxide formation. This oxidation introduces a new chiral center, resulting in the formation of two diastereomers, which are separated as distinct peaks after trypsin digestion. However, in the context of the full-length protein, this modification likely results in a single peak due to reduced chromatographic resolution (see peaks marked with asterisks in Figure 2b for representative examples).

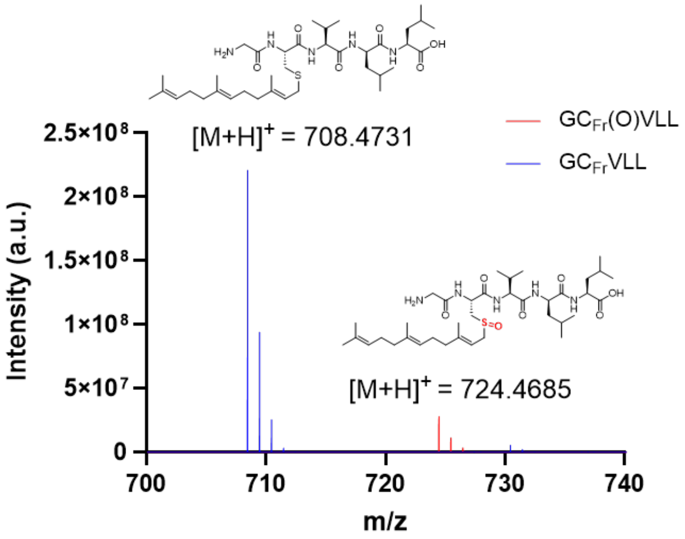

Figure S10. Overlaid mass spectra of the reduced and oxidized forms of the farnesylated (Fr)-modified C-terminal lipid fragments. A +16 Da mass shift is consistent with sulfoxide formation, as seen in the GG-modified peptide (Figure S9).

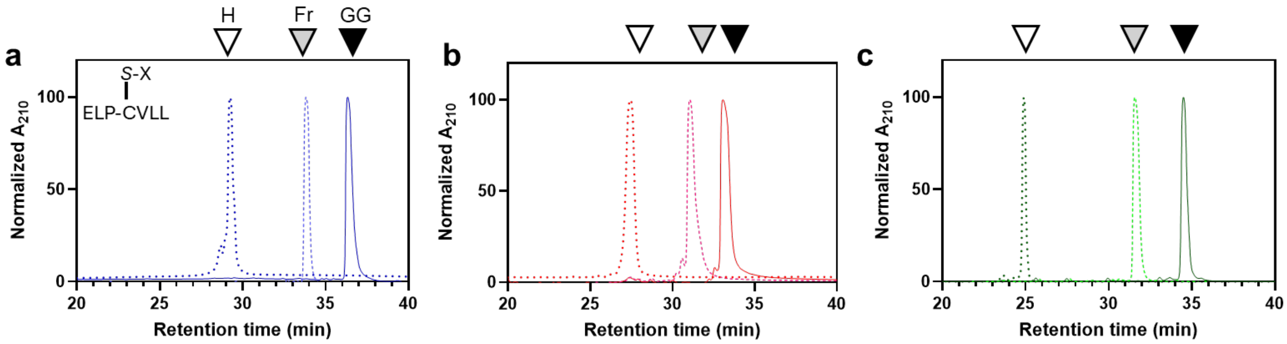

Figure S11. RP-HPLC traces of purified ELP isoforms used in this study. (a) ELPV40; **(b)** ELP(V8/A2)80; **(c)** ELPA40. In each panel, the unmodified isoform is represented by a dotted line, the Fr-modified isoform by a dashed line, and the GG-modified isoform by a solid line. Arrows indicate the retention times corresponding to each isoform.

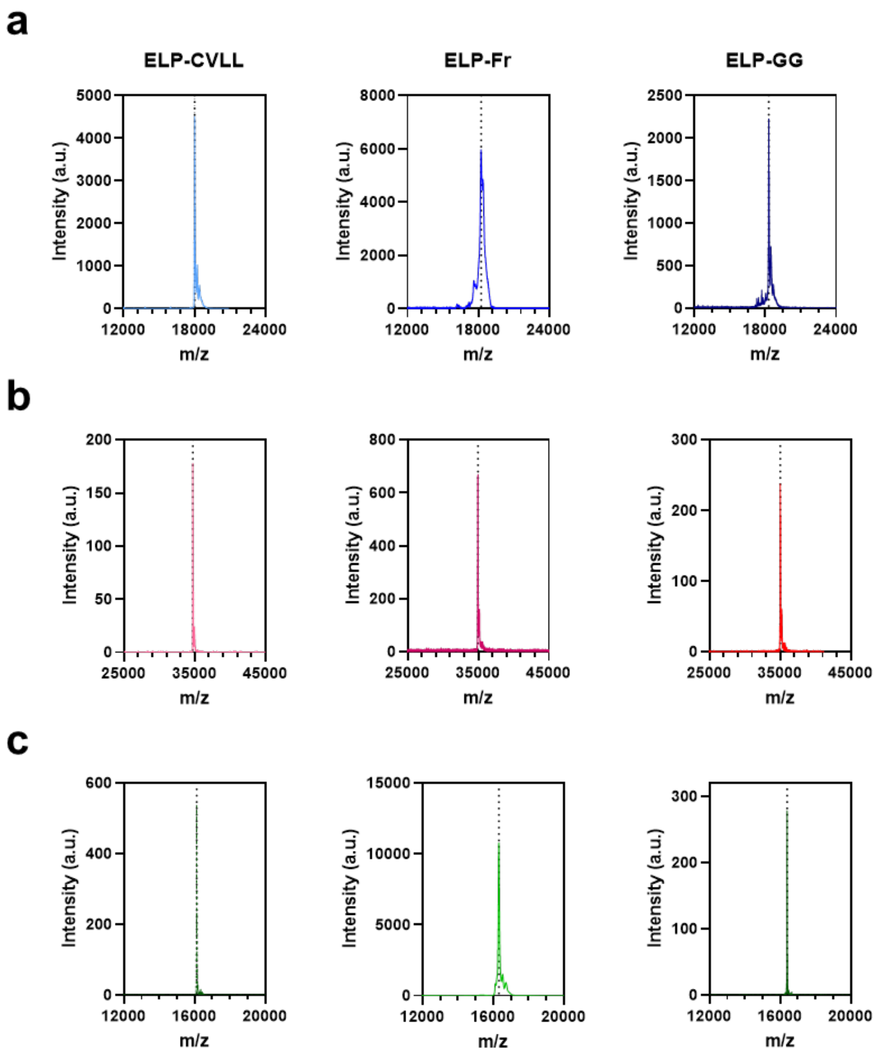

Figure S12. MALDI-TOF-MS spectra of the ELP isoforms in this study. (a) $ELPV_{40}$; **(b)** $ELP(V8/A2)_{80}$; **(c)** $ELPA_{40}$. Vertical dotted lines indicate the theoretical molecular weight ($[M + H]^+$) for each construct. See Table S7 for theoretical and observed masses.

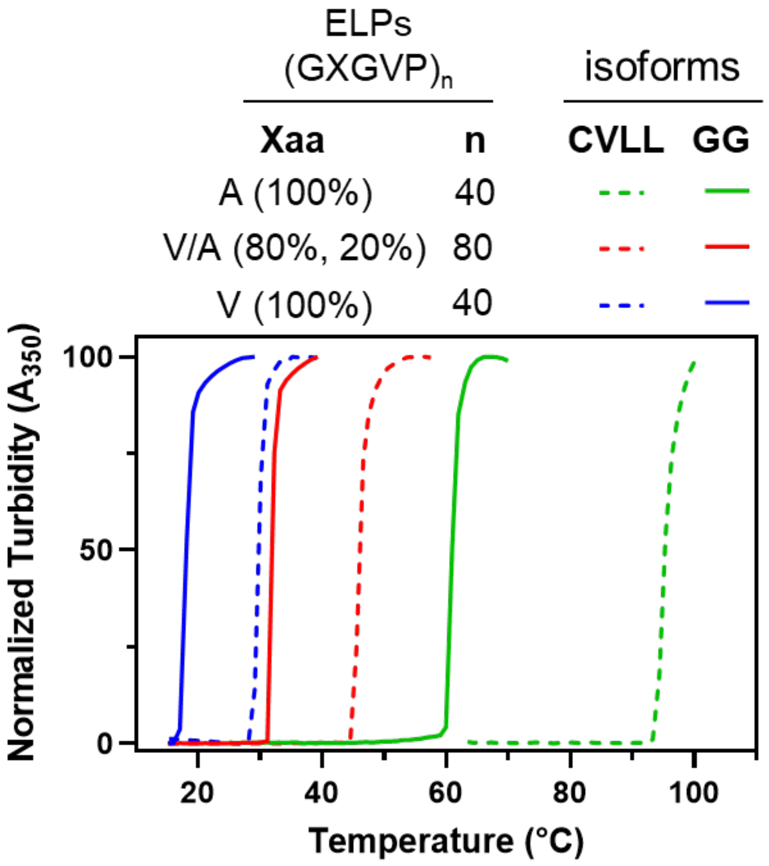

Figure S13. **Representative turbidimetry plots for geranylgeranylated and unmodified ELPs.** Geranylgeranylation increases the propensity of ELPs to phase-separate by lowering their transition temperature (T_t). Protein concentration = 50 μM in PBS.

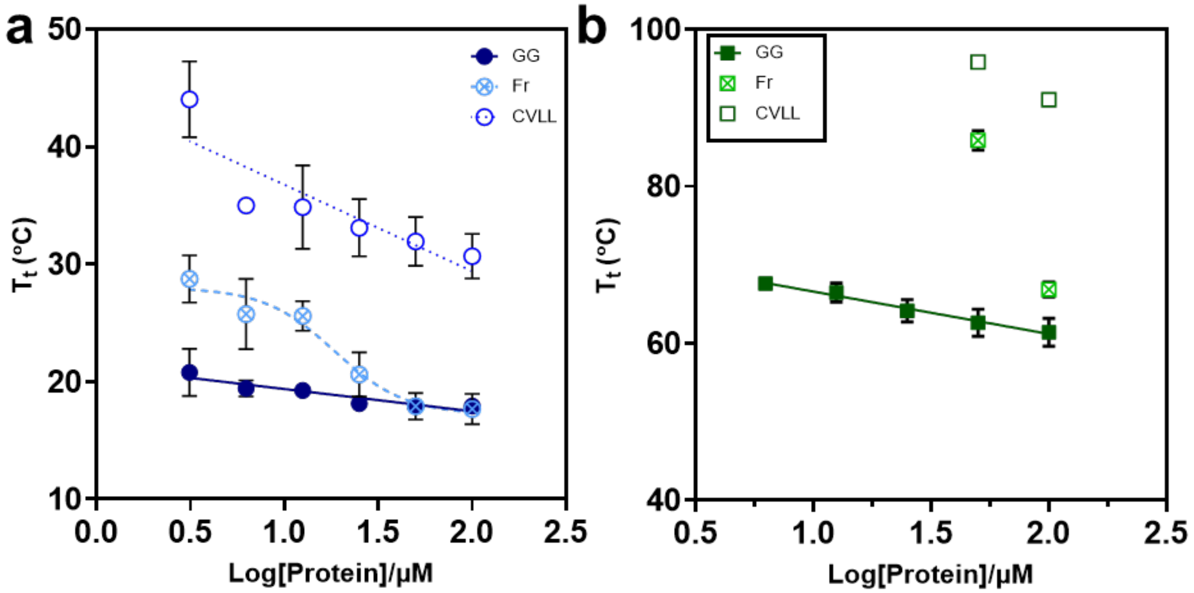

Figure S14. Temperature-composition plots for unmodified and prenylated ELPs. (a) ELPV$_{40}$: The transition temperature shows an inverse relationship with protein concentration. While the behavior of unmodified (dotted line) and GG-modified (solid line) ELPs is adequately described by linear regression, Fr-modified ELPs exhibit intermediate behavior, fitting a sigmoidal model (dashed line). **(b)** ELPA$_{40}$: Due to the hydrophilicity of these constructs, transition temperatures of unmodified and Fr-modified isoforms were outside the experimental range at concentrations below 50 μM. Data for GG-modified isoform is fitted to a linear model. Error bars represent the standard deviation of three measurements.

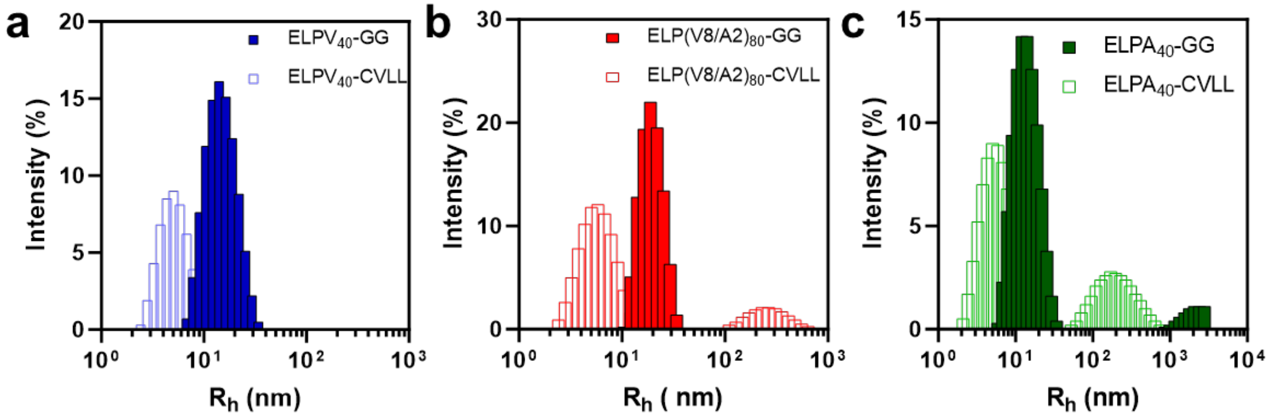

Figure S15. DLS intensity-size distributions of unmodified and geranylgeranylated ELP isoforms. **(a)** ELPV$_{40}$; **(b)** ELP(V8/A2)$_{80}$; **(c)** ELPA$_{40}$. In each case, GG-modification induces the self-assembly of ELPs into nanoparticles, as evidenced by increase in the diameter of the major peaks.

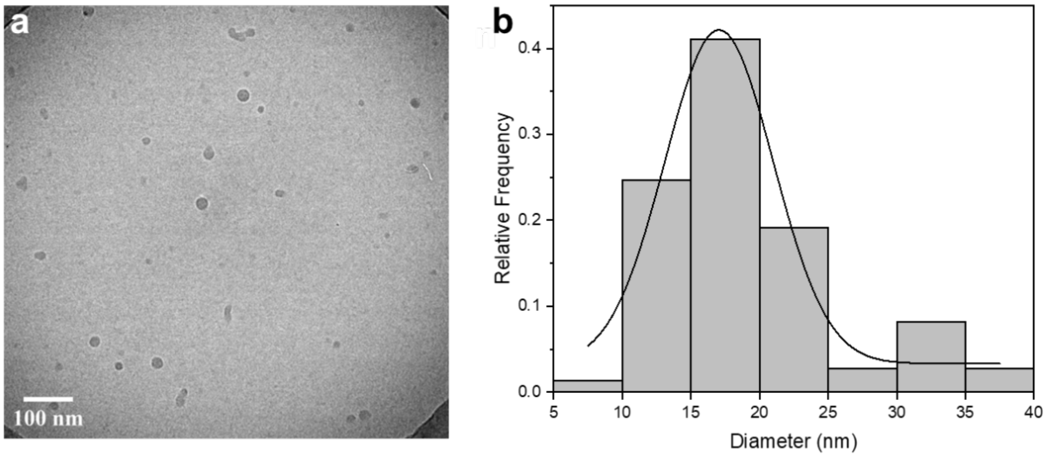

Figure S16. Cryo-TEM visualization of nanoparticles formed by ELP(V8/A2)$_{80}$-GG. (a) Representative cryo-TEM image of nanoparticles from samples vitrified at 25°C. **(b)** Histogram of nanoparticle diameter distributions.

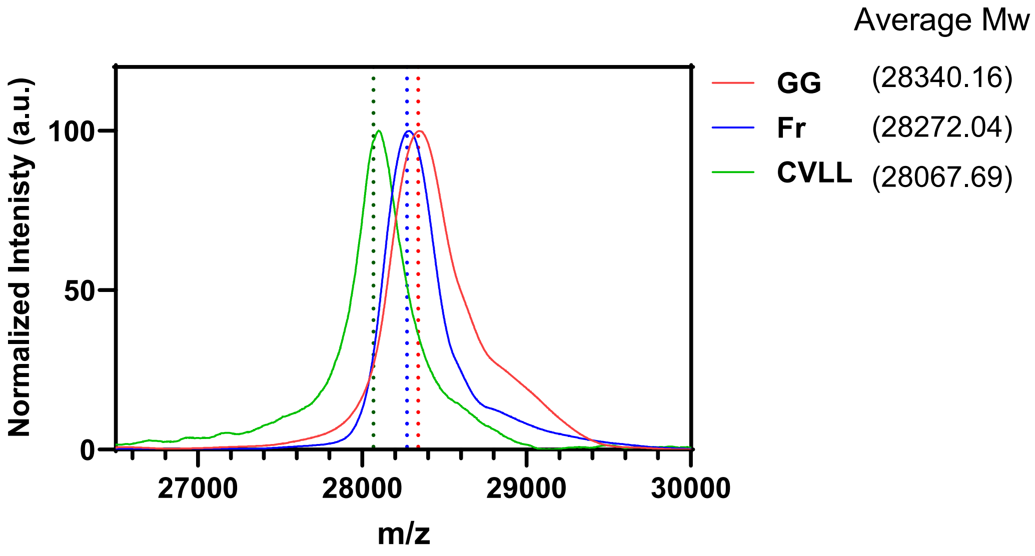

Figure S17. MALDI-TOF-MS analysis of mCherry isoforms. Vertical dotted lines denote the theoretical (average) molecular weights for each construct.

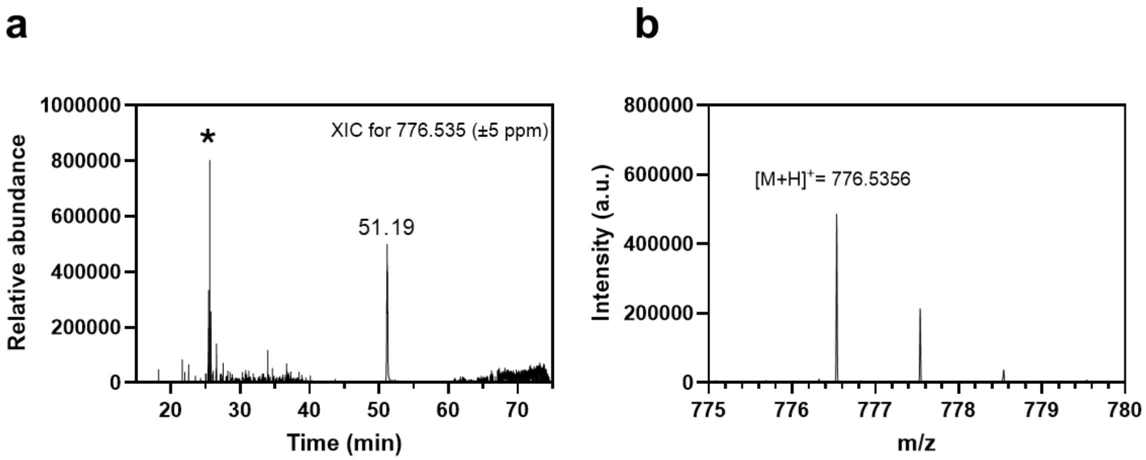

Figure S18. LC-MS characterization of the C-terminal fragments of mCherry-GG. (a) Extracted Ion Chromatogram (XIC) of trypsinized mCherry expressed in (+GGS/+GGT) strains shows a late-eluting peak at 51.19 minutes, with a molecular weight consistent with the geranylgeranylated 'GCVLL' peptide. **(b)** MS spectrum of this species displays mass accuracy within 0.7 ppm of the theoretical mass of $GC_{GG}VLL$ peptide. Peak marked with (*) is identified as noise based on the isotopic pattern analysis.

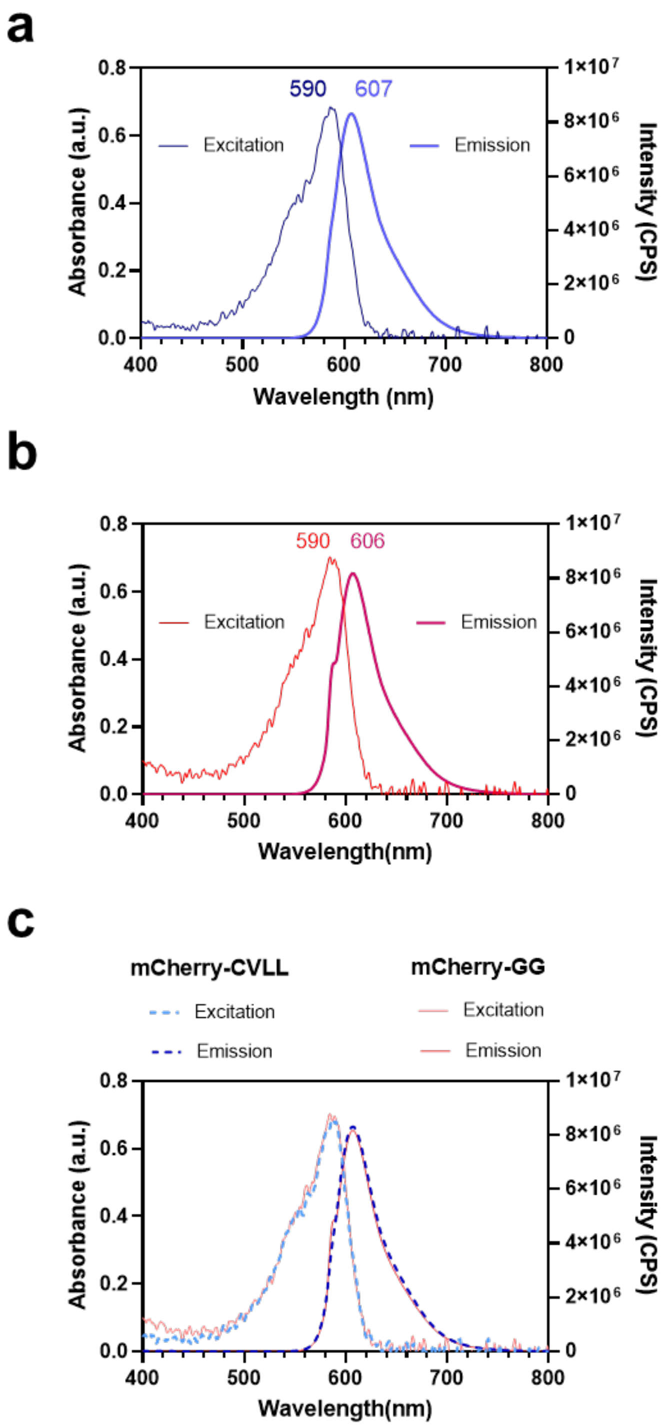

Figure S19. Excitation and emission spectra of unlipidated and GG-modified mCherry. (a) Unmodified isoform (mCherry-CVLL); (**b)** Geranylgeranylated isoform (mcherry-GG). Geranylgeranylation does not alter the fluorescence of mCherry; **(c)** Overlay of the excitation and emission spectra for both isoforms, demonstrating that the spectral properties remain unchanged upon geranylgeranylation.

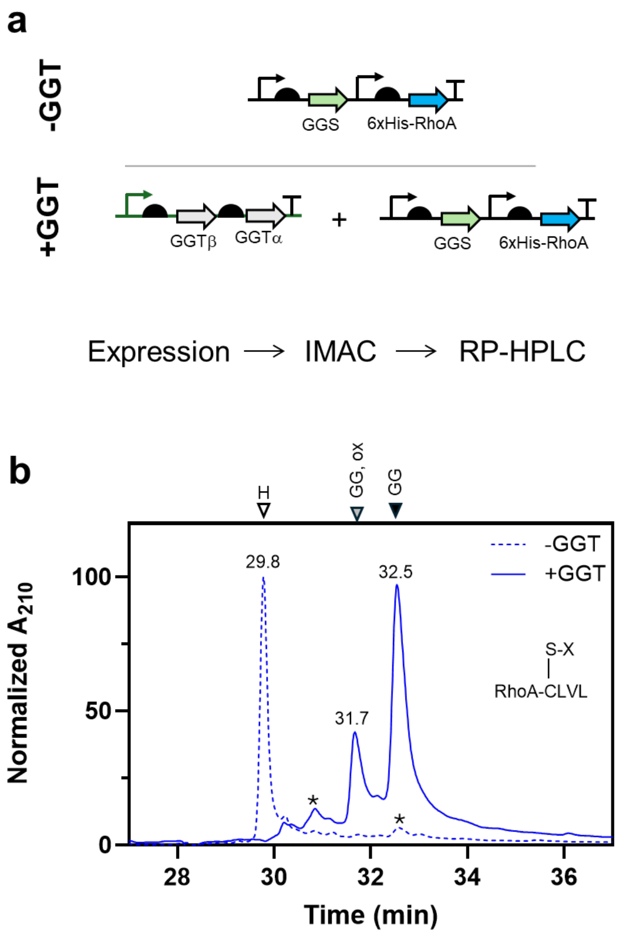

Condition	construct	t_R (min)	AUC	%
+GGT	n.d.*	30.8	208917	4.0
	GG-modified (Sulfoxide, GG,ox)	31.7	1146222	22.5
	GG-modified (GG)	32.5	3740751	73.5
-GGT	Unlipidated (H)	29.8	10411782	96.7
	n.d.*	32.5	353709	3.3

* n.d.: The peak identity is not determined.

Figure S20. Experimental workflow and analysis of recombinant production of GG-modified RhoA. (a) Schematic representation of the plasmids used for co-expression of RhoA with or without GGT. In both cases, RhoA is co-expressed with GGS. Since *E. coli* lacks endogenous GGT, a single plasmid is used for the -GGT condition. After expression, the soluble fraction was purified using immobilized metal affinity chromatography and analyzed by RP-HPLC. (b) RP-HPLC chromatogram comparing RhoA isoforms expressed with (solid line) and without (dashed line) GGT. In the absence of GGT, only unmodified RhoA is detected. Co-expression with GGT yields 96% geranylgeranylation (73.5% reduced, 22.5% sulfoxide). See Figures S22 and S24b for LC-MS analysis of each isoform.

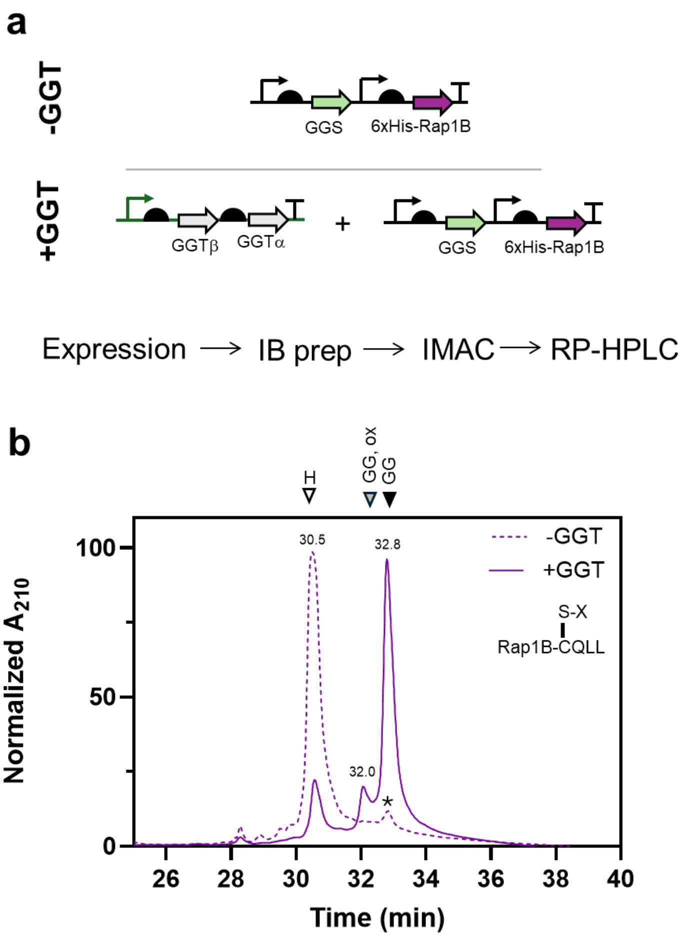

Condition	Construct	t_R (min)	AUC	%
+GGT	Unlipidated (H)	30.5	2470682	12.6
	GG-modified (Sulfoxide, GG,ox)	32.0	1030237	5.2
	GG-modified (GG)	32.8	16092669	82.2
- GGT	Unlipidated (H)	30.5	161626993	98.1
	n.d.*	32.8	1238780	1.9

* n.d.: The peak identity is not determined.

Figure S21. Experimental workflow and analysis of recombinant production of GG-modified Rap1B. (a) Schematic representation of the plasmids used for co-expression of Rap1B with or without GGT. In both cases, Rap1B is co-expressed with GGS. Since *E. coli* lacks endogenous GGT, a single plasmid is used for the -GGT condition. After expression, inclusion bodies were solubilized in 6M guanidine hydrochloride, purified by IMAC under denaturing condition, and analyzed by RP-HPLC analysis. (b) RP-HPLC chromatogram comparing Rap1B isoforms expressed with (solid line) and without (dashed line) GGT. In the absence of GGT, only unmodified Rap1B is detected. Co-expression with GGT yields 87% geranylgeranylation (82% reduced, 5% sulfoxide). See Figures S23 and S24a for LC-MS analysis of each isoform.

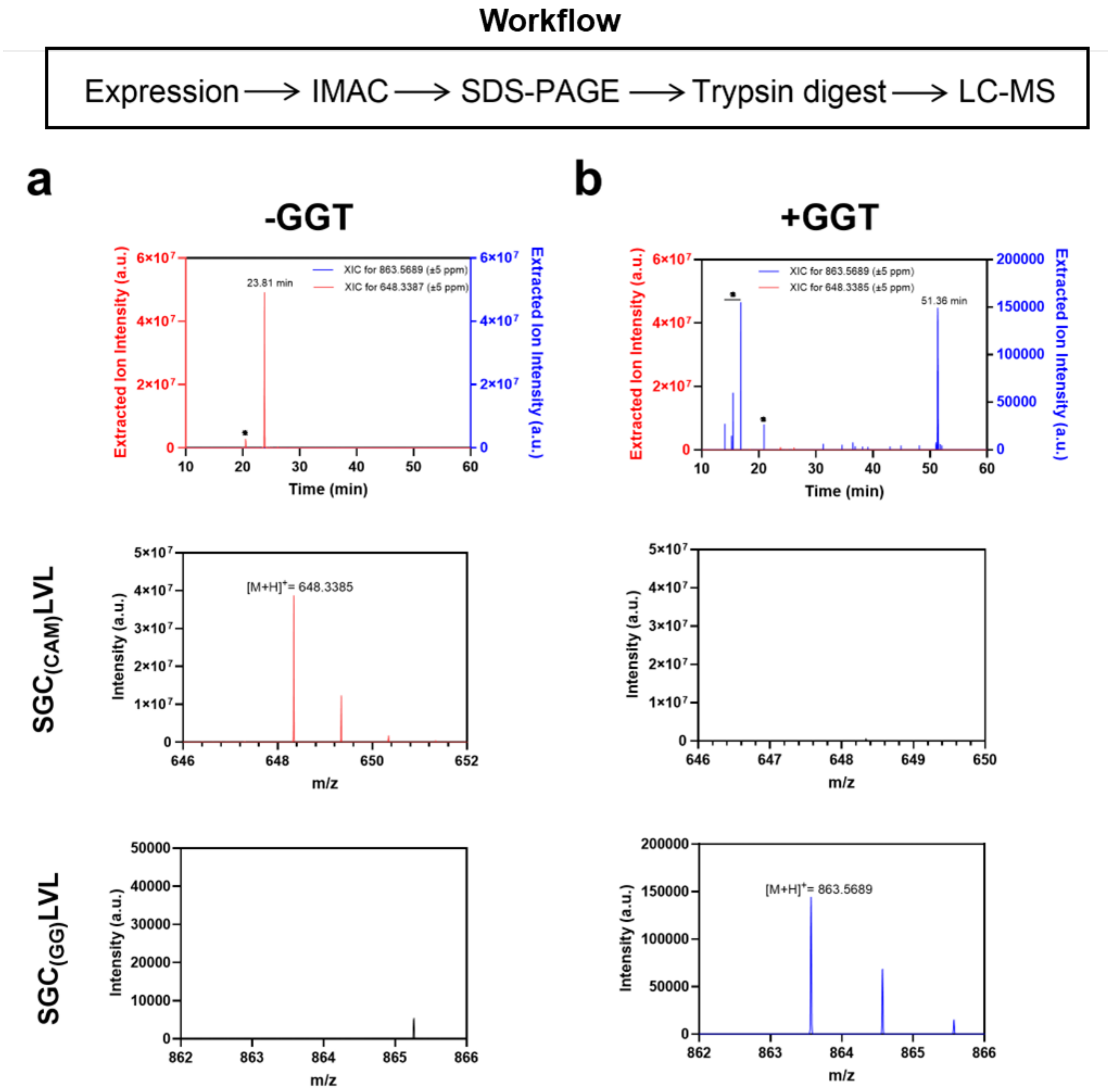

Figure S22. LC-MS analysis of the unmodified and geranylgeranylated C-terminal peptide fragment of RhoA. Extracted Ion Chromatogram (XIC) and mass spectra of trypsinized RhoA expressed in **(a)** -GGT (+GGS/-GGT) and **(b)** +GGT (+GGS/+GGT) strains. In the absence of GGT (panel a), only the unmodified C-terminal peptide fragment is detected at 23.81 min (red trace), while no GG-modified peptide is observed (blue trace). With GGT (panel b), a strong signal for the GG-modified fragment is detected (51.36 min), with minimal unmodified peptide present, consistent with RP-HPLC results (Figure S20). Free thiols in the unmodified constructs were alkylated using carboxymethylacetamide (Cam) to prevent dimerization. Peaks marked with (*) in the XIC trace were identified as noise based on the isotopic pattern analysis.

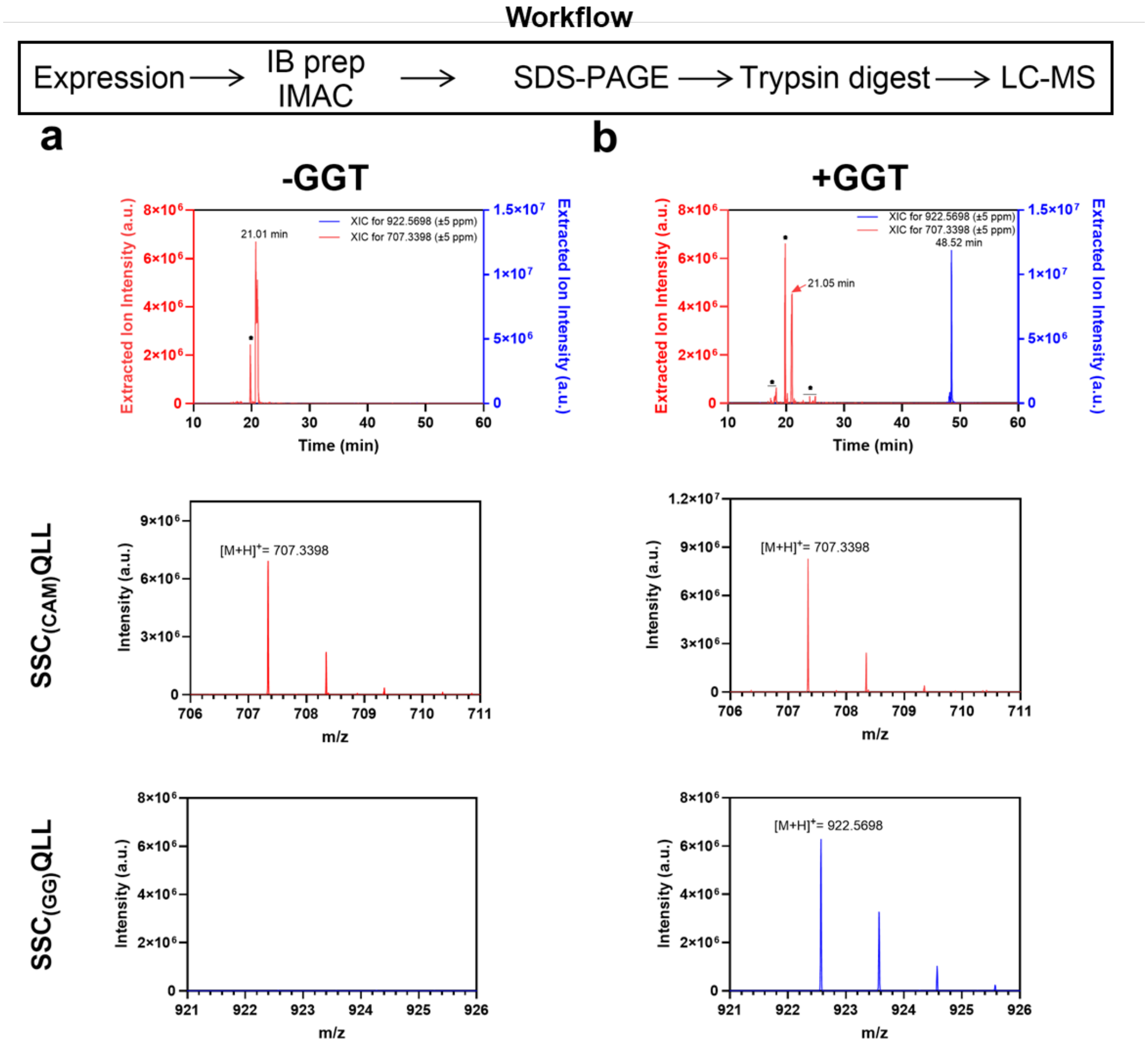

Figure S23. LC-MS analysis of the unmodified and geranylgeranylated C-terminal peptide fragment of Rap1B. Extracted Ion Chromatogram (XIC) and mass spectra of trypsinized Rap1B expressed in (a) -GGT (+GGS/-GGT) and (b) +GGT (+GGS/+GGT) strains. In the absence of GGT (panel a), only the unmodified C-terminal peptide fragment is detected at 21.01 min (red trace), with no GG-modified peptide observed (blue trace). Consistent with RP-HPLC results (Figure S21), under +GGT conditions (panel b), both unmodified (21.05 min) and GG-modified (48.52 min) peptides are detected. Free thiols in the unmodified constructs were alkylated with carboxymethylacetamide (Cam) to prevent dimerization. Peaks marked with (*) in the XIC trace were identified as noise based on isotopic pattern analysis.

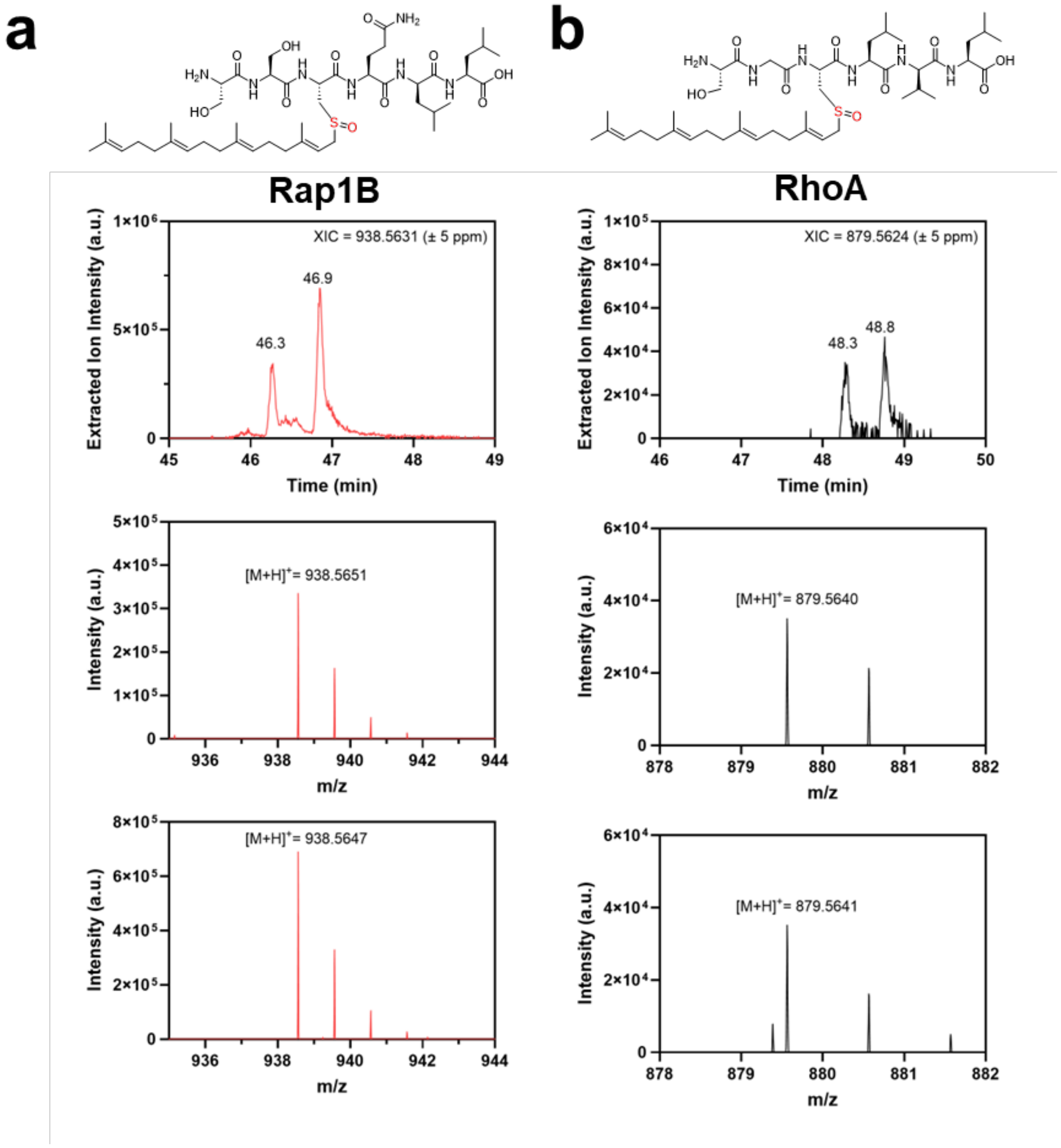

Figure S24. LC-MS analysis of the oxidized forms of the GG-modified C-terminal peptide fragments of Rap1B and RhoA. Chemical structures, extracted ion chromatograms, and mass spectra of C-terminal peptide fragments from model proteins expressed in (+GGS/+GGT) strain are shown for (a) Rap1B and (b) RhoA. In both cases, oxidation of the thioether bond in the GG-modified peptide results in two peaks eluting approximately 2–3 minutes earlier than the reduced GG-modified peak. The MS spectra confirm a +16 Da mass shift compared to the reduced form, consistent with sulfoxide formation. This oxidation introduces a new chiral center, resulting in the separation of two diastereomers as distinct peaks after trypsin digestion.

17. References

[1] M. Hossain, Z. Zhang, S. Ashok, A. Jenks, C. Lynch, J. Hougland, D. Mozhdehi, *ACS Appl. Bio Mater.* **2022**, *5*, 1846–1856.

[2] J. R. McDaniel, J. A. MacKay, F. G. Quiroz, A. Chilkoti, *Biomacromolecules* **2010**, *11*, 944–952.

[3] K. K. Zimmerman, J. D. Scholten, C. Huang, C. A. Fierke, D. J. Hupe, *Protein Expr. Purif.* **1998**, *14*, 395–402.

[4] J. Rappsilber, Y. Ishihama, M. Mann, *Anal. Chem.* **2003**, *75*, 663–670.

[5] C. Sweet, A. Aayush, L. Readnour, K. V. Solomon, D. H. Thompson, *Biomacromolecules* **2021**, *22*, 1990–1998.

[6] A. Micsonai, F. Wien, É. Bulyáki, J. Kun, É. Moussong, Y.-H. Lee, Y. Goto, M. Réfrégiers, J. Kardos, *Nucleic Acids Res.* **2018**, *46*, W315–W322.

[7] J. Abramson, J. Adler, J. Dunger, R. Evans, T. Green, A. Pritzel, O. Ronneberger, L. Willmore, A. J. Ballard, J. Bambrick, S. W. Bodenstein, D. A. Evans, C.-C. Hung, M. O'Neill, D. Reiman, K. Tunyasuvunakool, Z. Wu, A. Žemgulytė, E. Arvaniti, C. Beattie, O. Bertolli, A. Bridgland, A. Cherepanov, M. Congreve, A. I. Cowen-Rivers, A. Cowie, M. Figurnov, F. B. Fuchs, H. Gladman, R. Jain, Y. A. Khan, C. M. R. Low, K. Perlin, A. Potapenko, P. Savy, S. Singh, A. Stecula, A. Thillaisundaram, C. Tong, S. Yakneen, E. D. Zhong, M. Zielinski, A. Žídek, V. Bapst, P. Kohli, M. Jaderberg, D. Hassabis, J. M. Jumper, *Nature* **2024**, *630*, 493–500.

[8] E. Roberts, J. Eargle, D. Wright, Z. Luthey-Schulten, *BMC Bioinformatics* **2006**, *7*, 382.

[9] W. Humphrey, A. Dalke, K. Schulten, *J. Mol. Graph.* **1996**, *14*, 33–38.

[10] J.-M. Rondeau, F. Bitsch, E. Bourgier, M. Geiser, R. Hemmig, M. Kroemer, S. Lehmann, P. Ramage, S. Rieffel, A. Strauss, J. R. Green, W. Jahnke, *ChemMedChem* **2006**, *1*, 267–273.

[11] F. H. Wallrapp, J.-J. Pan, G. Ramamoorthy, D. E. Almonacid, B. S. Hillerich, R. Seidel, Y. Patskovsky, P. C. Babbitt, S. C. Almo, M. P. Jacobson, C. D. Poulter, *Proc. Natl. Acad. Sci.* **2013**, *110*, E1196–E1202.

[12] A. L. Marzinzik, R. Amstutz, G. Bold, E. Bourgier, S. Cotesta, J. F. Glickman, M. Götte, C. Henry, S. Lehmann, J. C. D. Hartwieg, S. Ofner, X. Pellé, T. P. Roddy, J.-M. Rondeau, F. Stauffer, S. J. Stout, A. Widmer, J. Zimmermann, T. Zoller, W. Jahnke, *ChemMedChem* **2015**, *10*, 1884–1891.

[13] J. C. Phillips, D. J. Hardy, J. D. C. Maia, J. E. Stone, J. V. Ribeiro, R. C. Bernardi, R. Buch, G. Fiorin, J. Hénin, W. Jiang, R. McGreevy, M. C. R. Melo, B. K. Radak, R. D. Skeel, A. Singharoy, Y. Wang, B. Roux, A. Aksimentiev, Z. Luthey-Schulten, L. V. Kalé, K. Schulten, C. Chipot, E. Tajkhorshid, *J. Chem. Phys.* **2020**, *153*, 044130.

[14] K. Vanommeslaeghe, E. Hatcher, C. Acharya, S. Kundu, S. Zhong, J. Shim, E. Darian, O. Guvench, P. Lopes, I. Vorobyov, A. D. Mackerell Jr., *J. Comput. Chem.* **2010**, *31*, 671–690.

[15] Y. T. Pang, Y. Miao, Y. Wang, J. A. McCammon, *J. Chem. Theory Comput.* **2017**, *13*, 9–19.

[16] T. Darden, D. York, L. Pedersen, *J. Chem. Phys.* **1993**, *98*, 10089–10092.

[17] J.-P. Ryckaert, G. Ciccotti, H. J. C. Berendsen, *J. Comput. Phys.* **1977**, *23*, 327–341.

[18] D. E. Meyer, A. Chilkoti, *Biomacromolecules* **2004**, *5*, 846–851.

[19] T. Ni, S. I. Williams, S. Rezelj, G. Anderluh, K. Harlos, P. J. Stansfeld, R. J. C. Gilbert, *Sci. Adv.* **2018**, *4*, eaaq0762.

[20] T. Baumgart, G. Hunt, E. R. Farkas, W. W. Webb, G. W. Feigenson, *Biochim. Biophys. Acta BBA - Biomembr.* **2007**, *1768*, 2182–2194.

[21] C. Liu, Z. Sun, S. Shen, L. Lin, T. Li, B. Tian, Y. Hua, *Lett. Appl. Microbiol.* **2014**, *58*, 219–224.
